# Diverse genetic pathways drive convergent fluconazole and caspofungin resistance phenotypes in *Candida albicans*

**DOI:** 10.1371/journal.pone.0360234

**Published:** 2026-10-09

**Authors:** Mounzer Abbas, Nour Fattouh, Christy Chedraoui, Setrida El Hachem, Tsolaire Sourenian, Ibrahim Bitar, Roy A. Khalaf

**Affiliations:** 1 Department of Biological Sciences, Lebanese American University, Byblos, Lebanon; 2 Department of Biology, Saint George University of Beirut, Beirut, Lebanon; 3 Department of Microbiology, Faculty of Medicine, University Hospital in Pilsen, Charles University, Pilsen, Czech Republic; 4 Biomedical Center, Faculty of Medicine, Charles University, Pilsen, Czech Republic; University of Chittagong, BANGLADESH

## Abstract

The emergence of antifungal resistance in *Candida albicans* is a major worldwide public health threat, particularly for immunocompromised patients. Antifungal resistance may arise through uniform resistance mechanisms or through variable evolutionary routes. The aim of this study was to investigate whether antifungal resistance arises in *C. albicans* through shared or isolate-specific mechanisms, in other words, following convergent or divergent adaptive pathways and genetic mechanisms. Six laboratory-induced fluconazole-resistant and six caspofungin-resistant isolates were analyzed using whole-genome sequencing coupled to phenotypic tests assessing pathogenicity characteristics. All fluconazole-resistant isolates showed increased ergosterol production and elevated ATP-binding cassette (ABC) transporter efflux pump activity, indicating conserved resistance adaptation mechanisms. Recurrent mutations included *ERG24* variants and *SOD6* S225P in all isolates while other mutations such as *ERG11* T128K varied between isolates. Caspofungin-resistant isolates exhibited diverse *CHS3* mutations causing variable chitin increases despite a shared resistance strategy. In addition, many isolates displayed decreased biofilm formation and virulence which suggests that increased resistance subjects virulence mechanisms to fitness trade-offs. Altogether, our laboratory-induced antifungal-resistant *C. albicans* isolates experienced convergent phenotypic adaptations that were the result of genetically diverse evolutionary routes. YMAP (Yeast Mapping Analysis Pipeline) observations also revealed large scale chromosomal rearrangements in our resistant isolates indicating that in addition to point mutations, rearrangements contribute to the acquisition of resistance. Our study highlights the complexity of resistance development and suggests that similar resistance outcomes may be driven by different genomic mechanisms.

## Introduction

Fungal infections pose a significant but underestimated global public health concern. Each year, around 6.5 million cases of invasive fungal infections are reported worldwide of which approximately 2.5 million result in mortality, especially in immunocompromised individuals such as HIV and cancer patients [1]. Among human fungal pathogens, species of the genus *Candida* represent the most common cause of fungal disease. Over 200 species of *Candida* exist but only five species: *Candida albicans*, *Candida glabrata*, *Candida krusei*, *Candida parapsilosis*, and *Candida tropicalis* cause 90% of *Candida*-associated infections worldwide [2]. Although an increasing trend towards isolation of non-*albicans Candida* species is globally observed [3], the opportunistic diploid *C. albicans* accounts for around 40–60% of invasive infections worldwide [4] and maintains high prevalence in some regions such as the Middle East and North Africa [5]. In Lebanon for example, *C. albicans* remains a common etiological agent of candidiasis, contributing substantially to vulvovaginal and bloodstream infections reported at tertiary care centers [6,7].

*C. albicans* infections are managed according to disease severity and site of infection. Superficial mucosal infections are usually treated by azole antifungals, particularly fluconazole while life-threatening invasive candidiasis and bloodstream infections rely on echinocandins, including anidulafungin, caspofungin, and micafungin [8]. Azoles inhibit the activity of lanosterol 14α-demethylase encoded by *ERG11*, thereby disrupting ergosterol biosynthesis and plasma membrane integrity. In contrast, echinocandins target the enzyme β-(1,3)-glucan synthase involved in fungal cell wall biosynthesis [9].

*C. albicans* are highly pathogenic as they are equipped with the ability to transition from yeast to filamentous forms including pseudohyphae and true hyphae [10]. The transition to true hyphae coupled to a strong adhesive capacity mediated by adhesins such as agglutinin-like sequence family proteins and Hwp1, promotes tissue invasion, biofilm formation, and immune interactions. Biofilms confer intrinsic tolerance to antifungals and promote the emergence of resistance. In parallel, *C. albicans* activates stress response pathways promoting survival in hostile host niches [11].

Although virulence remains a major clinical concern in the immunocompromised patients [12], an additional culprit is the presence of evidence indicating that *C. albicans* can acquire resistance to antifungals, particularly to fluconazole which is considered the first line of treatment [13]. Azole resistance is multifactorial, mainly involving target modifications, altered sterol biosynthesis, and increased drug efflux mediated by ABC and major facilitator superfamily transporters, often driven by gain-of-function mutations in key transcriptional regulators [14]. Echinocandin resistance is relatively low in prevalence but considered a dangerous occurrence since these antifungals are considered the last line of defense in life-threatening infections [15,16]. Echinocandin resistance is the result of point mutations in conserved hot-spot regions of *FKS* genes coding for β-(1,3)-glucan synthase [17]. These mutations could reduce fungal fitness but may be accompanied by compensatory cell wall remodeling mechanisms characterized by increased chitin deposition, contributing to increased resistance [18].

The aim of this study was to better understand the mechanisms underlying azole and echinocandin resistance in *C. albicans* by determining whether resistance acquired under a defined drug pressure arises through uniform genetic alterations across isolates or through diverse genetic routes that converge on similar resistance phenotypes. To address this question, we studied genomic and phenotypic changes in multiple colonies of a laboratory-induced antifungal-resistant *C. albicans* generated by sequential exposure to fluconazole or caspofungin at twice the minimum inhibitory concentration (MIC) from a previous study carried out in our research group by El Hachem et al. [19]. Through this approach, we were able to observe conserved resistance strategies driven by diverse underlying genetic changes.

## Results

### Whole-genome sequencing reveals extensive mutational heterogeneity across fluconazole and caspofungin-resistant isolates

Whole-genome sequencing identified substantial genomic diversity among resistant isolates with considerable variation in both the number and identity of mutations across isolates. No two resistant isolates shared identical mutational profiles (Tables 1 and 3). Despite this heterogeneity, several mutations were recurrent within resistance groups and accounted for the highest proportion of shared variants (Tables 1–4). In the fluconazole-resistant group, most isolates carried conserved mutations in the *ERG24* and *CHS3* genes involved in ergosterol biosynthesis and cell wall remodeling, respectively. In addition, a missense mutation leading to the S225P amino acid substitution in the oxidative stress response gene *SOD6* was observed in all fluconazole resistant isolates. Mutations associated with phenotypic switching and biofilm-related regulation were also recurrent, including variants in *MFG1*, *NAG3*, *UME6*, and *SFL1* (Table 2). In the caspofungin-resistant group, a limited set of recurrent mutations was detected. All isolates harbored the T486S substitution in the *GSC2* gene which codes for a catalytic subunit of β-(1,3)-glucan synthase. Additionally, S255P in Sod6 and L213S in Sir2 were frequently detected, supporting a role for oxidative stress adaptation and regulatory remodeling in caspofungin resistance (Table 4).

**Table 1:** Mutations detected in the targeted-genes in fluconazole-resistant isolates (FL1-FL6). This table groups all nonsynonymous mutations detected in isolates FL1 to FL6 compared to the reference strain in targeted genes associated with drug resistance, stress response, phenotypic switching and dimorphism, and biofilm formation.

Function	Gene	FL1	FL2	FL3	FL4	FL5	FL6
**Drug Resistance**	***ERG11***	T128K (M)/ E116D (M)		T128K (M)/ E116D (M)		E116D (M)	T128K (M)/ E116D (M)
	***MDR1***			I325V (M)	I325V (M)	I325V (M)	I325V (M)
	***ERG6***		I39V (M)			I39V (M)	
	***ERG24***	Q46H (M)	G119S (M)/ Q46H (M)	G119S (M)/ Q46H (M)	G119S (M)/ Q46H (M)	G119S (M)	G119S (M)/ Q46H (M)
	***CHS3***	P43L (M)/ *46* (SR)/ M74T (M)/ K94I (M)/ Q191R (M)/ T214I (M)/ D388G (M)/ R625Q (M)/ Q642R (M)/ *657L (SL)/ S682F (M)/ N686S (M)/ L708Q (M)/ R762P (M)/ G991E (M)	P43L (M)/ *46* (SR)/ M74T (M)/ K94I (M)/ Q191R (M)/ D388G (M)/ R625Q (M)/ Q642R (M)/ *657L (SL)/ S682F (M)/ N686S (M)/ L708Q (M)/ R762P (M)/ G991E (M)	P43L (M)/ *46* (SR)/ M74T (M)/ K94I (M)/ Q191R (M)/ T214I (M)/ P507L (M)/ R625Q (M)/ Q642R (M)/ *657L (SL)/ S682F (M)/ N686S (M)/ L708Q (M)/ G991E (M)	P43L (M)/ *46* (SR)/ M74T (M)/ K94I (M)/ Q191R (M)/ T214I (M)/ D388G (M)/ P507L (M)/ R625Q (M)/ Q642R (M)/ *657L (SL)/ S682F (M)/ N686S (M)/ L708Q (M)/ R762P (M)	P43L (M)/ *46* (SR)/ M74T (M)/ K94I (M)/ Q191R (M)/ T214I (M)/ P507L (M)/ R625Q (M)/ Q642R (M)/ *657L (SL)/ S682F (M)/ N686S (M)/ L708Q (M)/ R762P (M)/ G991E (M)	P43L (M)/ *46*(SR)/ M74T (M)/ K94I (M)/ Q191R (M)/ T214I (M)/ L321P (M)/ Q642R (M)/ *657L (SL)/ S682F (M)/ N686S (M)/ L708Q (M)
	***ERG4***		R18G (M)	M157V (M)			M157V (M)
**Stress Response**	***SOD5***			A210P (M)/ G214D (M)	A210P (M)/ G214D (M)	A210P (M)/ G214D (M)	A210P (M)
	***SOD6***	S225P (M)	S225P (M)	S225P (M)	S225P (M)	S225P (M)	S225P (M)
	***HSP60***		S267A (M)		S267A (M)	S267A (M)	S267A (M)
	***MNL1***	C170S (M)/ M43K (M)	C170S (M)/ I175T (M)/ R269* (SG)/ H349R (M)	M43K (M)	I175T (M)/ R269* (SG)/ H349R (M)/ A443V (M)	C170S (M)/ M43K (M)/ A443V (M)	I175T (M)/ R269* (SG)/ H349R (M)
**Phenotypic Switching/ Dimorphism**	***SAP1***	L53I (M)		L53I (M)	L53I (M)	L53I (M)	
	***CPH1***			T510A (M)		T510A (M)	Q579fs (F)
	***EFG1***		Q444_Q447del (DD)/ I395T (M)/ A392T (M)/ Q94del (DD)	Q94del (DD)/ Q444_Q447del (DD)	I395T (M)/ A392T (M)	I395T (M)/ A392T (M)	Q94del (DD)/ Q444_Q447del (DD)
	***MFG1***	N353S (M)/ S348N (M)/ L326Q (M)/ A296_Q297del (DD)/ Q281_A282insMAVQ (CI)/ I248M (M)/ T78_N79del (DD)/ A43G (M)	Q76_Q80del (CD)/ A68P (M)	Q76_Q80del (CD)/ A68P (M)/ N353S (M)/ S348N (M)/ Q328_Q329insQQQQ (DI)/ L326Q (M)/ A296_Q297del (DD)/ Q281_A282insMAVQ (CI)/ I248M (M)/ T78_N79del (DD)/ A43G (M)	N353S (M)/ S348N (M)/ Q328_Q329insQQQQ (DI)/ L326Q (M)/ A296_Q297del (DD)/ Q281_A282insMAVQ (CI)/ I248M (M)/ T78_N79del (DD)/ A43G (M)	Q76_Q80del (CD)/ A68P (M)/ N353S (M)/ S348N (M)/ Q328_Q329insQQQQ (DI)/ L326Q (M)/ A296_Q297del (DD)/ Q281_A282insMAVQ (CI)/ I248M (M)/ T78_N79del (DD)/ A43G (M)	A68P (M)/ N353S (M)/ S348N (M)/ Q328_Q329insQQQQ (CI)/ L326Q (M)/ A296_Q297del (DD)/ Q281_A282insMAVQ (CI)/ I248M (M)/ T78_N79del (DD)/ A43G (M)
	***SIR2***	L213S (M)	K235R (M)/ D171_S174del (CD)	K235R (M)	K235R (M)	K235R (M)	L213S (M)
	***RFG1***	N188fs (F)/ Q130del (CD)	Q506del (CD)/ G319_G320dup (CI)/ N194dup (DI)/ Q130del (CD)	Q130del (CD)/ T51S (M)	T51S (M)	T51S (M)	T51S (M)
	***FGR15***	S361L (M)	S361L (M)/ V131A (M)			V131A (M)	
**Biofilm Formation**	***HWP1***	S441P (M)/ T296A (M)	S441P (M)		S492P (M)	S492P (M)	S492P (M)
	***ALS4***	A43V (M)/ I79V (M)/ V83I (M)/ V111A (M)/ S186P (M)/ D198N (M)	I79V (M)/ V83I (M)/ V111A (M)/ S186P (M)/ D198N (M)	I79V (M)/ V83I (M)/ V111A (M)/ S186P (M)/ D198N (M)	I79V (M)/ V83I (M)/ V111A (M)/ S186P (M)/ D198N (M)	S111F (M)/ LI147ST (M)/ Q151P (M)/ K130E (M)	A43V (M)/ S111F (M)/ LI147ST (M)/ Q151P (M)/ K130E (M)
	***NAG3***	A397V (M)	A397V (M)		A397V (M)	A397V (M)	A397V (M)
	***UME6***	M44L (M)/ Q76_Q77dup (DI)/ N257S (M)/ M267I (M)/ N396S (M)/ N484del (CD)/ E81K (M)	N257S (M)/ M267I (M)/ D43_H46del (DD)/ G55D (M)/ M78T (M)/ V82I (M)	D43_H46del (DD)/ G55D (M)/ M78T (M)/ V82I (M)	Q76_Q77dup (DI)/ N257S (M)/ M267I (M)/ N396S (M)/ N484del (CD)/ E81K (M)	N257S (M)/ N396S (M)/ N484del (CD)/ E81K (M)	N257S (M)/ M267I (M)/ N396S (M)/ D43_H46del (DD)/ G55D (M)/ M78T (M)/ V82I (M)
	***SFL1***	S373L (M)/ Q301fs (F)	S373L (M)/ T75A (M)	P735T (M)/ V691I (M)/ S373L (M)/ Q302_Q304del (DD)/ T75A (M)	V691I (M)/ T75A (M)	P735T (M)/ S373L (M)/ T75A (M)	P735T (M)/ V691I (M)/ S373L (M)/ T75A (M)

Abbreviations for variant type: M, missense; F, frameshift; SR, stop retained; SL, stop lost; SG, stop gained; DI, disruptive in-frame insertion; DD, disruptive in-frame deletion; CI, conservative in-frame insertion; CD, conservative in-frame deletion. Other abbreviations: *, stop codon; fs, frameshift (for example, Q579fs means that the frameshift starts at Q579); del, deletion (for example, Q444_Q447del means that deletion starts at Q444 and ends at Q447 while Q94del means that only Q94 was deleted); ins, insertion (for example, Q281_A282insMAVQ means that MAVQ were inserted between Q281 and A282); dup, duplication (for example, Q76_Q77dup means that Q at positions 76 and 77 were duplicated).

**Table 2:** Recurrent mutations identified in fluconazole-resistant isolates (FL1-FL6). Nonsynonymous mutations that have occurred in at least 5 of 6 isolates are shown along with their frequency across isolates.

Gene	Mutation	Frequency (%)	FL1	FL2	FL3	FL4	FL5	FL6
***ERG24***	Q46H (M)	83.3	X	X	X	X	–	X
***ERG24***	G119S (M)	83.3	–	X	X	X	X	X
***CHS3***	P43L (M)	100	X	X	X	X	X	X
***CHS3***	L708Q (M)	100	X	X	X	X	X	X
***CHS3***	*46* (SR)	100	X	X	X	X	X	X
***CHS3***	Q642R (M)	100	X	X	X	X	X	X
***CHS3***	S682F (M)	100	X	X	X	X	X	X
***CHS3***	R625Q (M)	83.3	X	X	X	X	X	–
***CHS3***	M74T (M)	100	X	X	X	X	X	X
***CHS3***	*657L (SL)	100	X	X	X	X	X	X
***CHS3***	Q191R (M)	100	X	X	X	X	X	X
***CHS3***	T214I (M)	83.3	X	–	X	X	X	X
***CHS3***	N686S (M)	100	X	X	X	X	X	X
***CHS3***	K94I (M)	100	X	X	X	X	X	X
***SOD6***	S225P (M)	100	X	X	X	X	X	X
***MFG1***	Q281_A282insMAVQ (CI)	83.3	X	–	X	X	X	X
***MFG1***	L326Q (M)	83.3	X	–	X	X	X	X
***MFG1***	I248M (M)	83.3	X	–	X	X	X	X
***MFG1***	N353S (M)	83.3	X	–	X	X	X	X
***MFG1***	T78_N79del (DD)	83.3	X	–	X	X	X	X
***MFG1***	S348N (M)	83.3	X	–	X	X	X	X
***MFG1***	A296_Q297del (DD)	83.3	X	–	X	X	X	X
***MFG1***	A43G (M)	83.3	X	–	X	X	X	X
***NAG3***	A397V (M)	83.3	X	X	–	X	X	X
***UME6***	N257S (M)	83.3	X	X	–	X	X	X
***SFL1***	S373L (M)	83.3	X	X	X	–	X	X

Abbreviations for variant type: M, missense; SR, stop retained; SL, stop lost; DD, disruptive in-frame deletion; CI, conservative in-frame insertion. Other abbreviations: *, stop codon; del, deletion (for example, T78_N79del means that deletion starts at T78 and ends at N79); ins, insertion (for example, Q281_A282insMAVQ means that MAVQ were inserted between Q281 and A282). Detection notation: X, variant detected; -, variant not detected.

**Table 3:** Mutations detected in the targeted-genes in caspofungin-resistant isolates (CS1-CS6). This table groups all nonsynonymous mutations detected in isolates CS1 to CS6 compared to the reference strain in targeted genes associated with drug resistance, stress response, phenotypic switching and dimorphism, and biofilm formation.

Function	Gene	CS1	CS2	CS3	CS4	CS5	CS6
**Drug Resistance**	***MDR1***	I325V (M)			I325V (M)		
	***SSD1***			P43H (M)/ P80L (M)/ N96S (M)/ L128Q (M)	N10dup (DI)/ S161P (M)/ G173A (M)/ T179N (M)	N10dup (DI)/ S161P (M)/ G173A (M)/ T179N (M)/ P43H (M)/ P80L (M)/ N96S (M)/ L128Q (M)	S161P (M)/ G173A (M)/ T179N (M)/ P43H (M)/ P80L (M)/ N96S (M)/ L128Q (M)
	***CRZ2***			S334A (M)		S334A (M)	T26A (M)/ T58A (M)/ TS64IG (M)/ P151_Q152insQQQ (CI)/ A179T (M)/ S334A (M)
	***GSC2***	T486S (M)	T486S (M)	T486S (M)/ A438P (M)	T486S (M)	T486S (M)	T486S (M)/ A438P (M)
	***CHS3***	L321P (M)/ R762P (M)/ G991E (M)	P43L (M)/ *46* (SR)/ M74T (M)/ K94I (M)/ Q191R (M)/ T214I (M)/ P507L (M)/ R625Q (M)/ Q642R (M)/ *657L (SL)/ S682F (M)/ N686S (M)/ L708Q (M)	P43L (M)/ *46* (SR)/ M74T (M)/ K94I (M)/ Q191R (M)/ T214I (M)/ L321P (M)/ D388G (M)/ R625Q (M)/ Q642R (M)/ *657L (SL)/ S682F (M)/ N686S (M)/ L708Q (M)/ R762P (M)/ G991E (M)	L321P (M)/ P507L (M)	D388G (M)	L321P (M)/ D388G (M)/ R762P (M)/ G991E (M)
**Stress Response**	***SOD5***		G214D (M)	A210P (M)/ G214D (M)	A210P (M)/ G214D (M)		A210P (M)/ G214D (M)
	***SOD6***	S225P (M)	S225P (M)	S225P (M)	S225P (M)	S225P (M)	
	***HSP60***			S267A (M)			S267A (M)
	***MNL1***	C170S (M)/ H349R (M)/ A443V (M)	C170S (M)	M43K (M)	R269* (SG)	C170S (M)/ H349R (M)	M43K (M)/ I175T (M)/ R269* (SG)/ H349R (M)/ A443V (M)
	***ATG26***	E269K (M)	S43L (M)/ T414I (M)/ K470dup (DI)	T414I (M)/ E235K (M)/ K203R (M)/ E164K (M)	S43L (M)/ E269K (M)/ Y401C (M)	E269K (M)/ T414I (M)	E269K (M)/ Y401C (M)/ K470dup (DI)/ E235K (M)/ T206_T207insAT (CI)/ K203R (M)/ E164K (M)
**Phenotypic Switching/ Dimorphism**	***CPH1***		T656A (M)/ V627D (M)/ Q579_Q580del (DD)/ T510A (M)	T656A (M)/ V627D (M)/ Q579_Q580del (DD)/ T510A (M)		T656A (M)/ V627D (M)/ Q579_Q580del (DD)	T656A (M)/ V627D (M)
	***MFG1***		A68P (M)/ Q76_Q80del (CD)		A43G (M)/ T78_N79del (DD)	N353S (M)/ S348N (M)/ Q328_Q329insQQQQ (DI)/ L326Q (M)/ A296_Q297del (DD)/ Q281_A282insMAVQ (CI)/ I248M (M)/ T78_N79del (DD)/ A43G (M)	Q76_Q80del (CD)/ N353S (M)/ S348N (M)/ L326Q (M)
	***RFG1***	T51S (M)	Q130del (CD)	T51S (M)	Q506del (CD)/ G319_G320dup (CI)/ N194dup (DI)/ Q130del (CD)	Q506del (CD)/ Q130del (CD)	Q506del (CD)/ G319_G320dup (CI)/ N194dup (DI)/ Q130del (CD)
	***SIR2***	L213S (M)/ H11R (M)/ D171_S174del (CD)	L213S (M)	K235R (M)	L213S (M)	L213S (M)	L213S (M)
	***FGR15***	V131A (M)	S361L (M)	V131A (M)	V131A (M)		
**Biofilm Formation**	***HWP1***	S492P (M)	S441P (M)/ T296A (M)	S441P (M)	S441P (M)	S492P (M)	S492P (M)/ S441P (M)
	***ALS4***	S111F (M)/ L147_I148delinsST (M)/ Q151P (M)/ K130E (M)	I79V (M)/ V83I (M)/ V111A (M)/ S186P (M)/ D198N (M)	I79V (M)/ V83I (M)/ V111A (M)/ S186P (M)/ D198N (M)	S111F (M)/ LI147ST (M)/ Q151P (M)/ K130E (M)	A43V (M)/ G65S (M)/ I79V (M)/ V83I (M)/ V111A (M)/ S186P (M)/ D198N (M)	I79V (M)/ V83I (M)/ V111A (M)
	***NAG3***	A397V (M)		A397V (M)	A397V (M)	A397V (M)	
	***UME6***	M44L (M)/ Q76_Q77dup (DI)/ N257S (M)/ M267I (M)/ N484del (CD)/ E81K (M)	N396S (M)/ D43_H46del (DD)/ G55D (M)/ M78T (M)/ V82I (M)	N257S (M)/ M267I (M)/ D43_H46del (DD)/ G55D (M)/ M78T (M)/ V82I (M)	M44L (M)/ Q76_Q77dup (DI)/ N484del (CD)/ E81K (M)	M44L (M)/ Q76_Q77dup (DI)/ M267I (M)/ N396S (M)/ N484del (CD)/ E81K (M)	M44L (M)/ Q76_Q77dup (DI)/ N257S (M)/ N484del (CD)/ D43_H46del (DD)/ G55D (M)/ M78T (M)/ V82I (M)
	***SFL1***	S373L (M)		P735T (M)/ V691I (M)/ Q302_Q304del (DD)/ T75A (M)	V691I (M)	P735T (M)/ V691I (M)/ S373L (M)/ Q302_Q304del (DD)/ T75A (M)	Q302_Q304del (DD)

Abbreviations for variant type: M, missense; F, frameshift; SR, stop retained; SL, stop lost; SG, stop gained; DI, disruptive in-frame insertion; DD, disruptive in-frame deletion; CI, conservative in-frame insertion; CD, conservative in-frame deletion. Other abbreviations: *, stop codon; del, deletion (for example, Q579_Q580del means that deletion starts at Q579 and ends at Q580 while Q130del means that only Q130 was deleted); ins, insertion (for example, P151_Q152insQQQ means that QQQ were inserted between P151 and Q152); dup, duplication (for example, G319_G320dup means that G at positions 319 and 320 were duplicated while N10dup means that N at position 10 was duplicated); delins, deletion followed by insertion resulting in amino acid replacement (for example, L147_I148delinsST means that L147 and I148 were deleted and replaced by S and T at positions 147 and 148, respectively).

**Table 4:** Recurrent mutations identified in caspofungin-resistant isolates (CS1-CS6). Nonsynonymous mutations that have occurred in at least 5 of 6 isolates are shown along with their frequency across isolates.

Gene	Mutation	Frequency (%)	CS1	CS2	CS3	CS4	CS5	CS6
***GSC2***	T486S (M)	100	X	X	X	X	X	X
***SOD6***	S225P (M)	83.3	X	X	X	X	X	–
***SIR2***	L213S (M)	83.3	X	X	–	X	X	X

Abbreviations for variant type: M, missense. Detection notation: X, variant detected; -, variant not detected.

### Resistant isolates cluster by antifungal pressure despite heterogeneous mutational landscapes

Average nucleotide identity (ANI) heat maps were generated to assess genomic relatedness between drug-resistant isolates and the reference strain. All fluconazole-resistant isolates showed decreased ANI values compared to the reference strain but maintained similar ANI values among each other (Fig 1). A similar trend was observed for caspofungin-resistant isolates. CS6 showed slightly lower similarity compared to isolates CS1 to CS5 (Fig 2). Overall, resistant isolates were more closely related to one another than to the reference strain (Fig 3).

**Fig 1:**
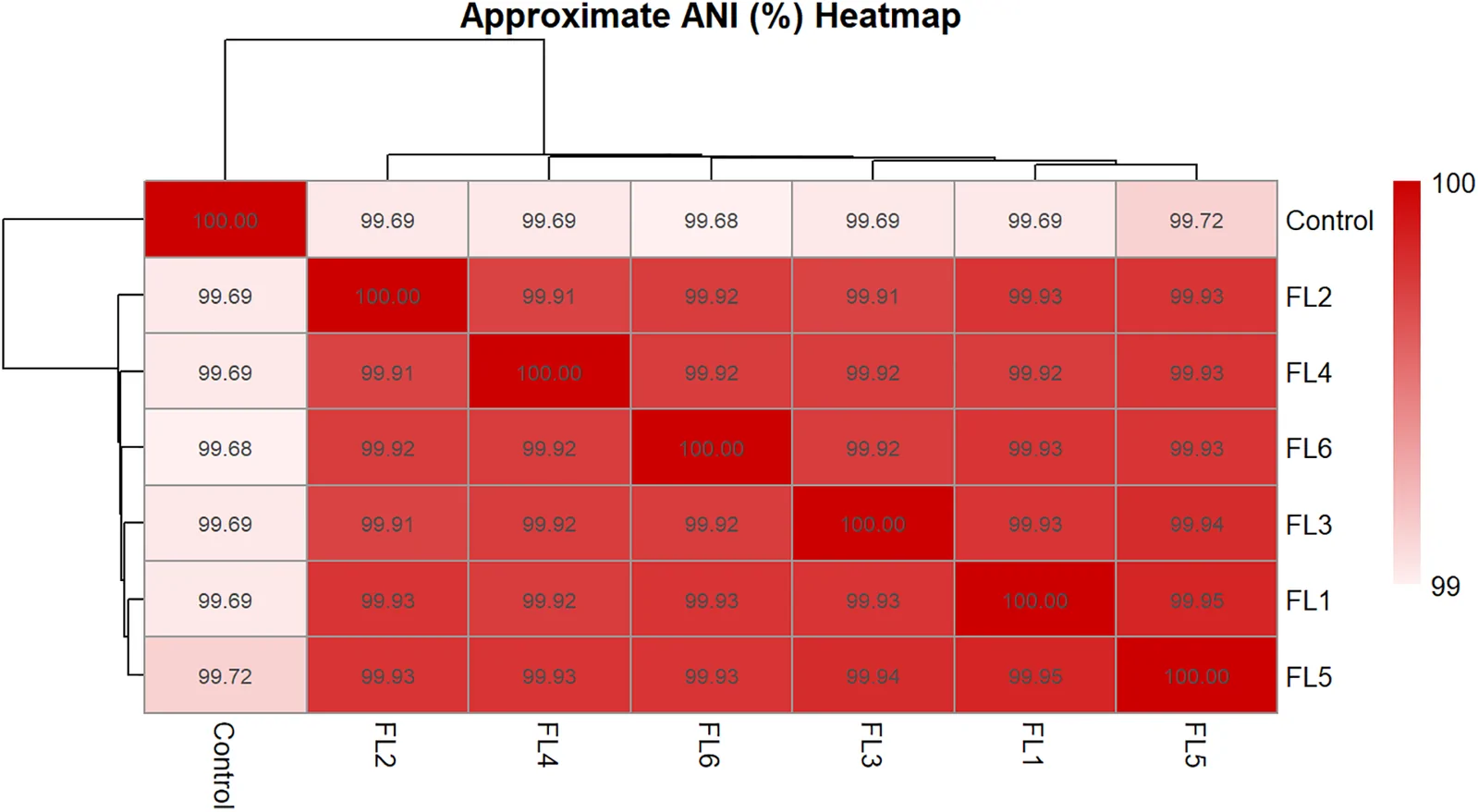
Average nucleotide identity (ANI) heatmap showing genome-wide similarity among the fluconazole-resistant isolates (FL1-FL6) and the reference strain *C. albicans* SC5314 (control). ANI values were estimated from Mash distances and represent approximate genome-wide similarity based on shared *k*-mers rather than exact nucleotide identity. The heatmap and hierarchical clustering are descriptive visualizations of genomic relatedness, with lower ANI values indicating greater genomic divergence.

**Fig 2:**
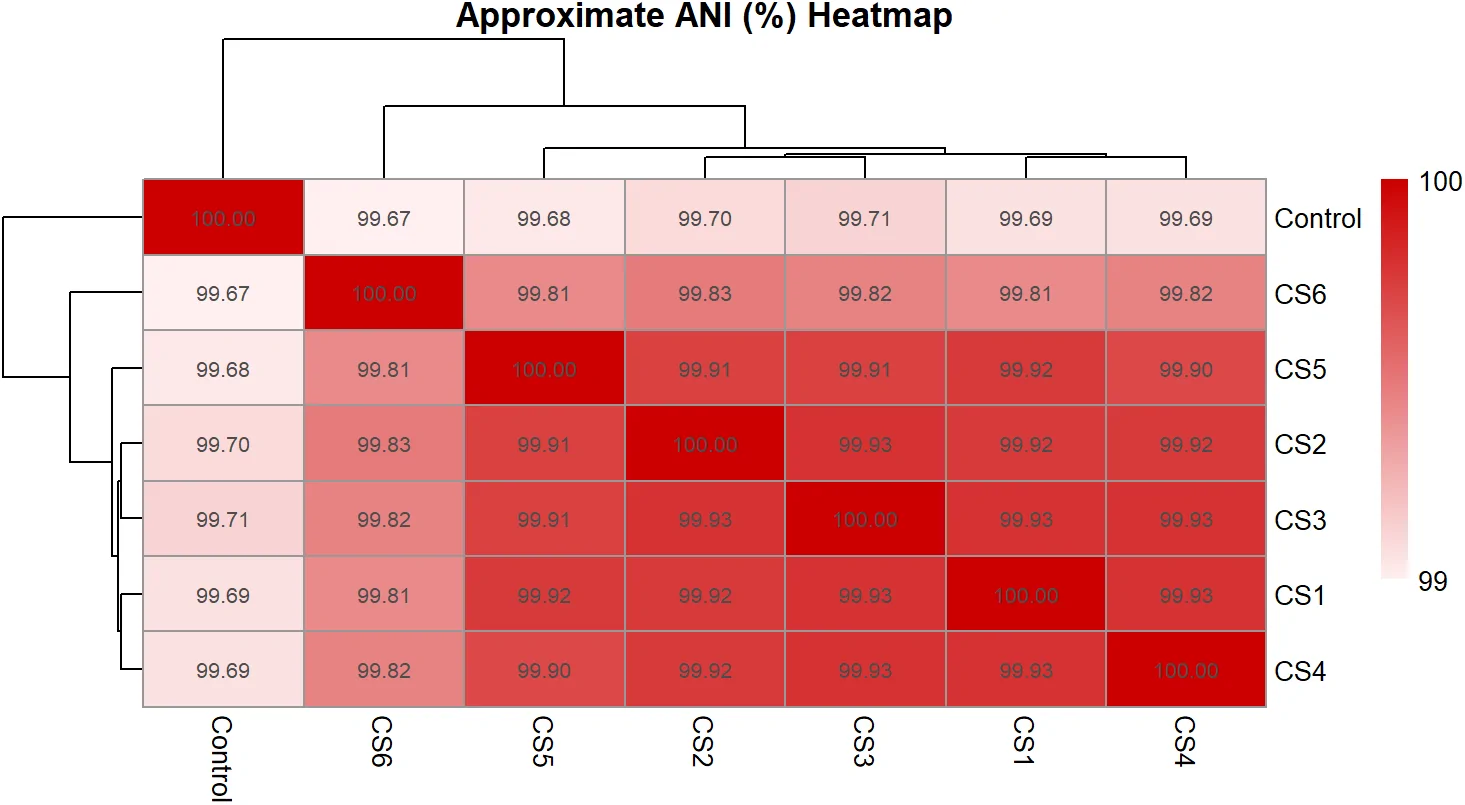
Average nucleotide identity (ANI) heatmap showing genome-wide similarity among the caspofungin-resistant isolates (CS1-CS6) and the reference strain *C. albicans* SC5314 (control). ANI values were estimated from Mash distances and represent approximate genome-wide similarity based on shared *k*-mers rather than exact nucleotide identity. The heatmap and hierarchical clustering are descriptive visualizations of genomic relatedness, with lower ANI values indicating greater genomic divergence.

**Fig 3:**
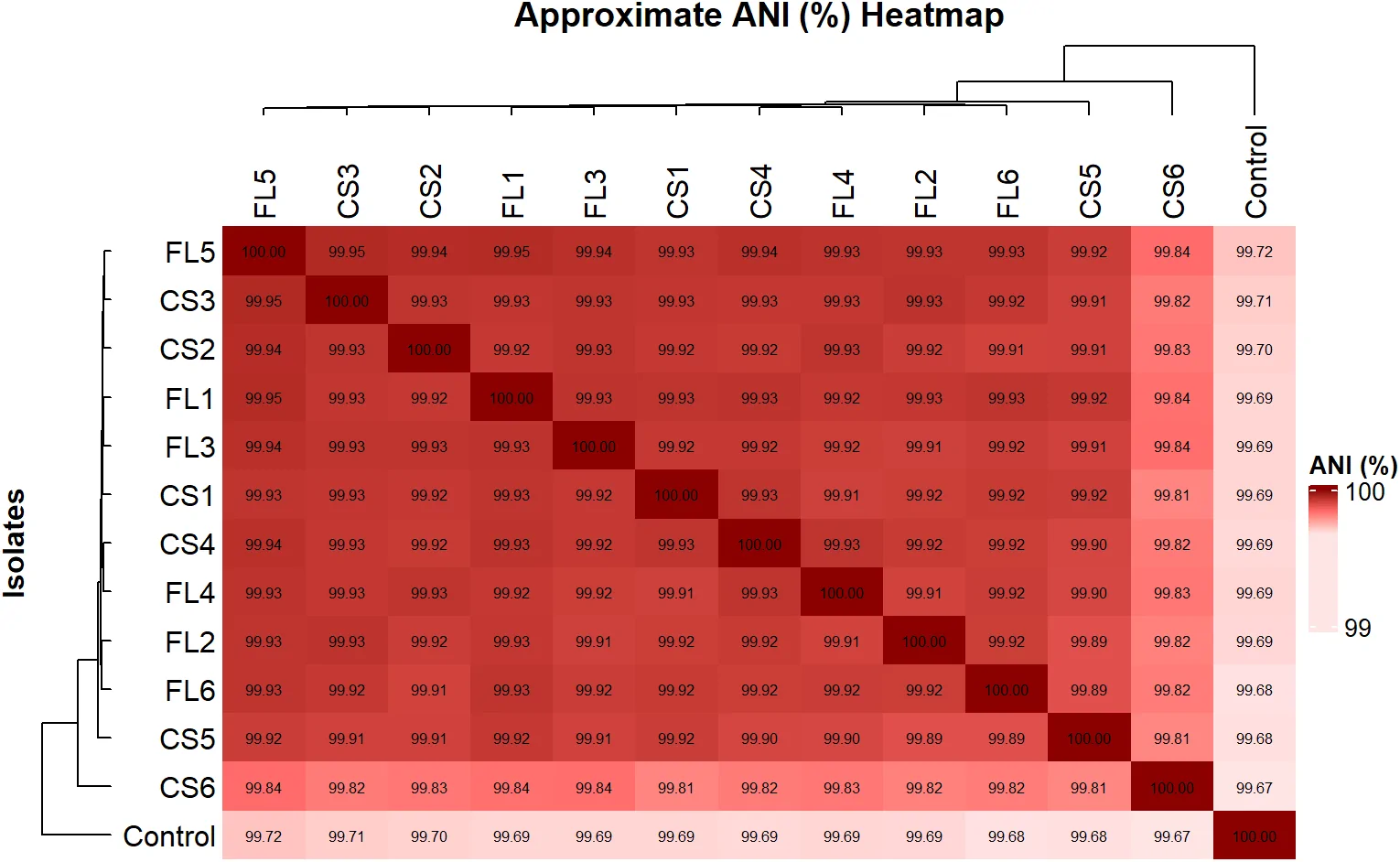
Average nucleotide (ANI) heatmap showing genome-wide similarity among fluconazole-resistant (FL1-FL6) and caspofungin-resistant (CS1-CS6) isolates and the reference strain *C. albicans* SC5314 (control). ANI values were estimated from Mash distances and represent approximate genome-wide similarity based on shared *k*-mers rather than exact nucleotide identity. The heatmap and hierarchical clustering are descriptive visualizations of genomic relatedness, with lower ANI values indicating greater genomic divergence.

### Murine survival analysis reveals isolate-dependent virulence differences

Virulence was assessed using a murine infection model for fluconazole and caspofungin-resistant isolates. During the first 3 days post-infection, one moribund mouse was observed in the control group infected by the reference strain as well as the groups infected by isolates FL3 and FL6. Over the one month period, survival outcomes differed substantially among isolates. Groups infected by FL1 and FL5 showed the highest survival with 5 of 6 mice alive, followed by FL3 with 4 of 6 mice alive. Groups injected by FL2, FL4, CS1, and CS2 each exhibited 3 mice surviving, while the group injected with the reference strain, FL6, and CS4 had 2 mice that survived 30 days. The groups infected by CS5 and CS6 each had one surviving mouse and CS3 exhibited complete mortality. Overall, fluconazole-resistant isolates generally exhibited reduced virulence compared to the reference strain, with 64% survival, while caspofungin-resistant isolates displayed variable virulence profiles, with an average of 28% survival (Fig 4).

**Fig 4:**
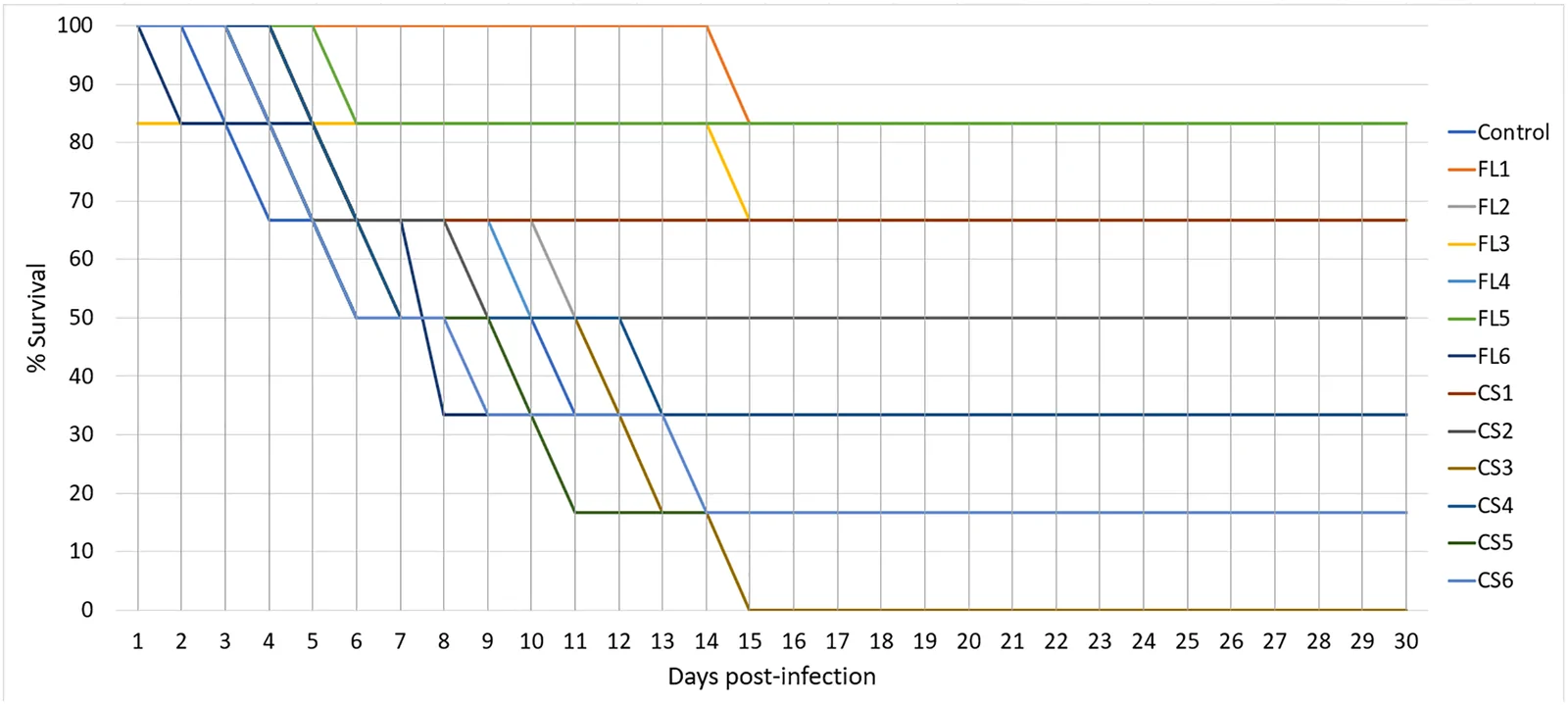
Heatmap of daily morbidity in a murine model of disseminated *C. albicans* infection. The heatmap shows the number of days vertically and the *C. albicans* isolates injected each in 6 mice horizontally. Each cell is color coded according to the number of moribund mice identified on a daily basis and which is represented by the color scale. FL1-FL6 are the fluconazole-resistant isolates while CS1-CS6 are the caspofungin-resistant isolates. The control group was infected by the reference strain *C. albicans* SC5314.

### Fluconazole and caspofungin-resistant isolates exhibit increased ergosterol content

A significant increase in ergosterol content was observed in the fluconazole and caspofungin-resistant groups of isolates compared to the control. In the fluconazole-resistant group of isolates, FL4, FL5, and FL6 showed the most pronounced increase since they contained 81%, 77%, and 71% higher ergosterol content in comparison to the control. More modest increases in ergosterol content were observed in FL1, FL2, and FL3. In the caspofungin-resistant group, all isolates experienced an increase in ergosterol content ranging from 28% in CS4–67% in CS2 (Fig 5). Overall, the consistent elevation of ergosterol content across both resistance groups indicates phenotypic convergence toward altered membrane sterol composition despite genetic heterogeneity among isolates. The increase in ergosterol quantity in the group of fluconazole-resistant isolates FL1 to FL6 (Fig 5, white bars) and caspofungin-resistant isolates CS1 to CS6 (Fig 5, grey bars) compared to the control is statistically significant with respective p-values of 0.0130 and 0.0138.

**Fig 5:**
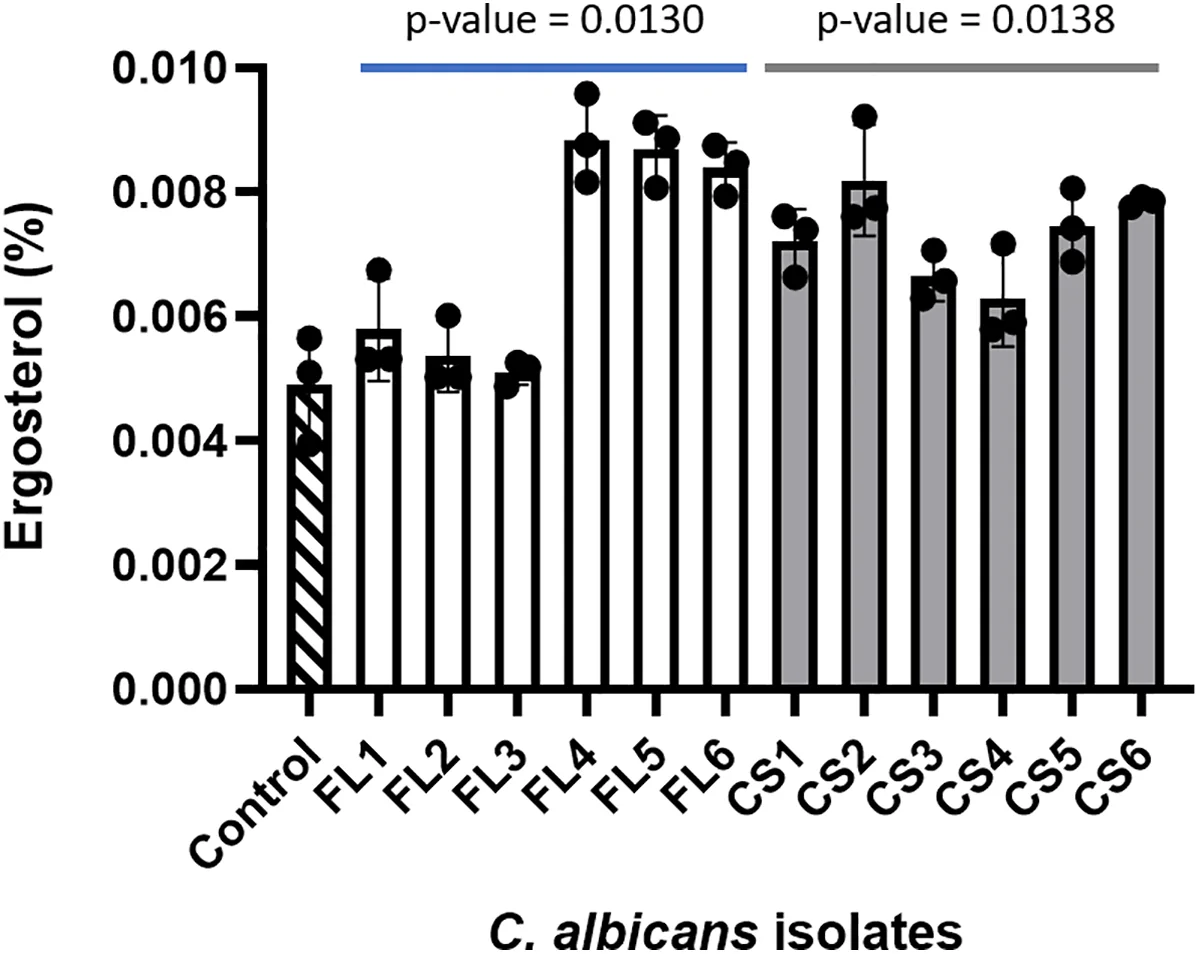
Ergosterol content in fluconazole (FL1-FL6) and caspofungin-resistant (CS1-CS6) *C. albicans* isolates. Ergosterol content is expressed as a percentage of total sterols normalized to biomass weight. The reference strain *C. albicans* SC5314 was used as control and its quantity of ergosterol is represented by the striped bar. The p-values for the fluconazole-resistant and caspofungin-resistant groups of isolates relative to the control are shown.

### Fluconazole and caspofungin-resistant isolates exhibit increased cell wall chitin content

A significant increase in cell wall chitin content was observed in the fluconazole and caspofungin-resistant groups of isolates compared to the control. In the fluconazole-resistant group of isolates, FL1, FL2, and FL6 showed the most pronounced increases. More modest increases in chitin content were observed in FL3, FL4, and FL5. Caspofungin-resistant isolates experienced a strong chitin accumulation supporting the compensatory ability of cell wall remodeling under echinocandin pressure. CS1 showed the strongest increase in chitin content among all studied isolates, while CS2 exhibited the lowest increase of 8%. The remaining caspofungin-resistant isolates CS3, CS4, CS5, and CS6 also showed marked increases in chitin content of 74%, 77%, 87%, and 100%, respectively (Fig 6). Overall, increased chitin synthesis seems to be a shared adaptive response associated with antifungal resistance, particularly in caspofungin-resistant strains. The increase in chitin concentration in the group of fluconazole-resistant isolates FL1 to FL6 (Fig 6, white bars) and caspofungin-resistant isolates CS1 to CS6 (Fig 6, grey bars) compared to the control is statistically significant with respective p-values of 0.0057 and 0.0006.

**Fig 6:**
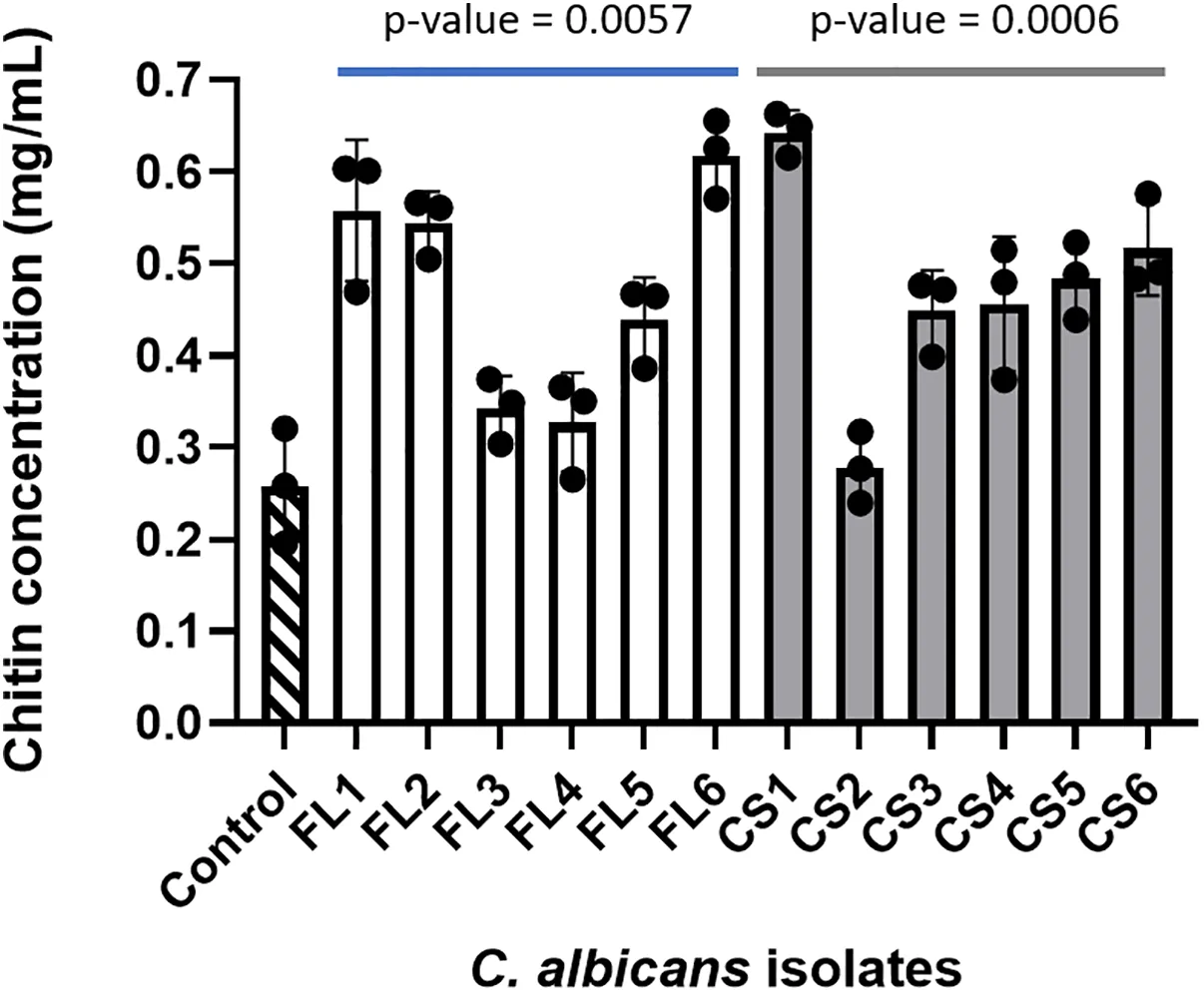
Chitin content in fluconazole (FL1-FL6) and caspofungin-resistant (CS1-CS6) *C. albicans* isolates. Chitin concentration in mg/mL of 100 mg biomass wet weight pellet were used. The reference strain *C. albicans* SC5314 was used as control and its chitin concentration is represented by the striped bar. The p-values for the fluconazole-resistant and caspofungin-resistant groups of isolates relative to the control are shown.

### Most fluconazole-resistant isolates exhibit increased ABC transporters efflux activity

Rhodamine 6G (R6G) was quantified as a proxy for ABC efflux pump activity, particularly the activity of Cdr1(Candida drug resistance 1) and Cdr2 (Candida drug resistance 2). All fluconazole-resistant isolates experienced a statistically significant increase in efflux activity except for FL2 that exhibited a slight reduction (Fig 7). Overall, these results indicate that enhanced ABC transporter activity is a frequent phenotype among our fluconazole-resistant isolates. FL1, FL3, FL4, FL5, and FL6 experienced a significant increase in R6G efflux with respective p-values of 0.0021, 0.0013, < 0.0001, 0.0003, and 0.0014.

**Fig 7:**
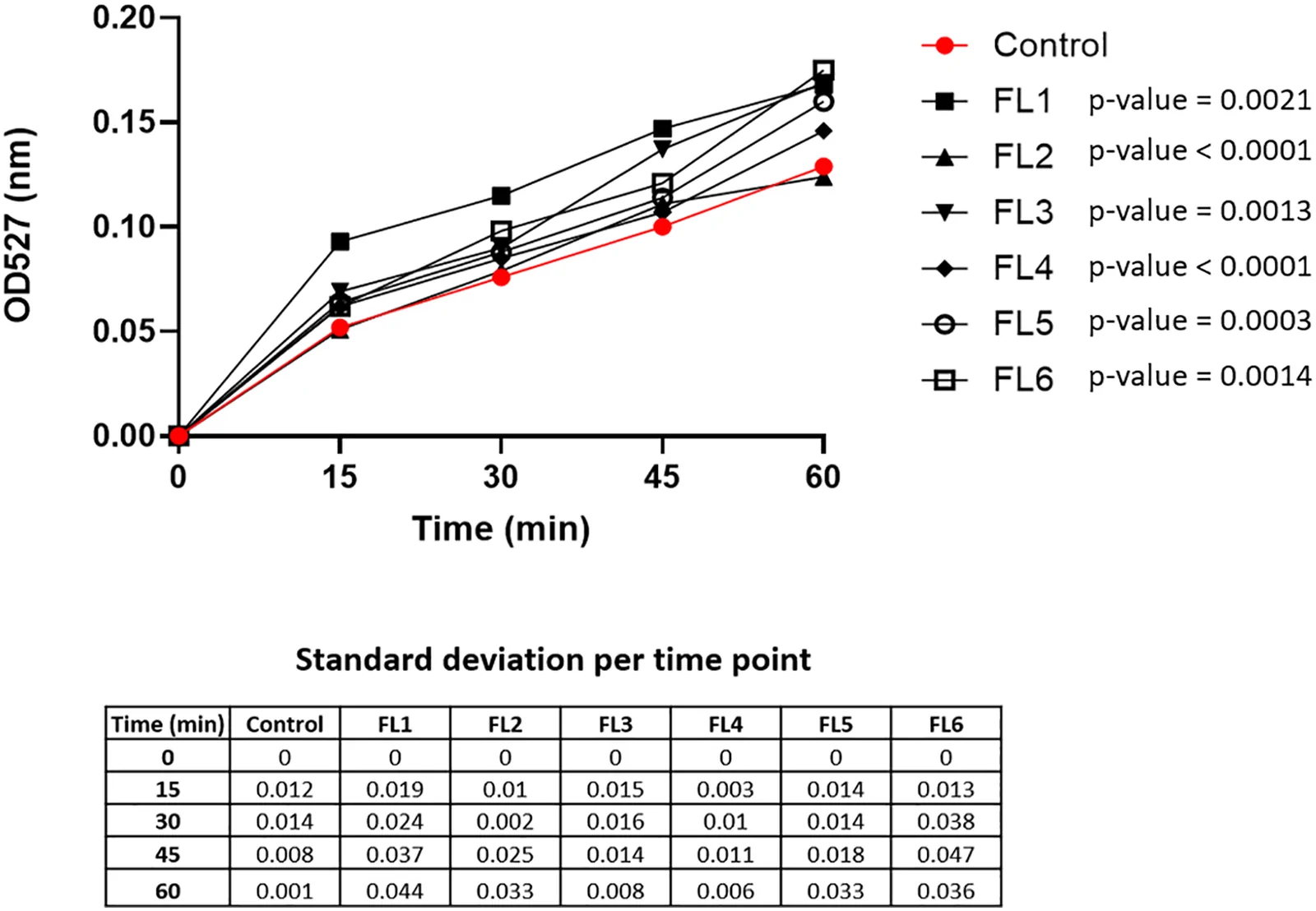
R6G efflux activity as a measure of ABC transporter activity in fluconazole-resistant *C. albicans* isolates. The reference strain *C. albicans* SC5314 was used as control and R6G efflux in function of time is represented by the red curve. The changes in R6G efflux were quantified by measuring OD at 527 nm over time in each fluconazole-resistant isolate (FL1 to FL6; black curves differentiated by distinct symbols) compared to the control and the differences were statistically significant. Each data point represents the mean value of three replicates. The standard deviation for each data point is shown in the table. The p-values for each isolate relative to the control are shown.

### Fluconazole and caspofungin-resistant isolates experience reduced biofilm formation

A significant decrease in biofilm formation was observed in the fluconazole and caspofungin-resistant groups of isolates compared to the control. Among fluconazole-resistant isolates, reductions in biofilm formation ranged from 75% in FL2–26% in FL6 and among caspofungin-resistant isolates, reductions varied from 66% in CS6–26% in CS2 (Fig 8). Overall, these results indicate that acquisition of antifungal resistance is associated with impaired biofilm-forming capacity. The decrease in biofilm biomass in the group of fluconazole-resistant isolates FL1 to FL6 (Fig 8, white bars) and caspofungin-resistant isolates CS1 to CS6 (Fig 8, grey bars) compared to the control is statistically significant with respective p-values of 0.0104 and 0.0061 (Fig 9).

**Fig 8:**
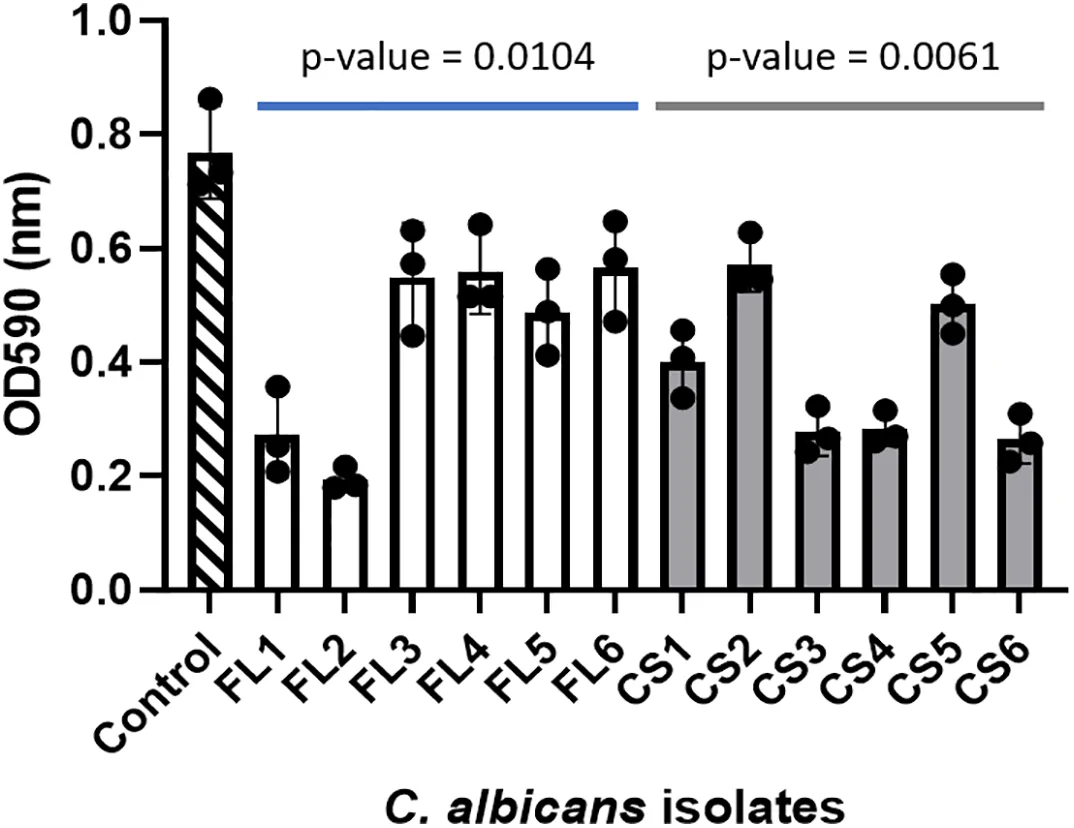
Biofilm biomass production in fluconazole (FL1-FL6) and caspofungin-resistant (CS1-CS6) *C. albicans* isolates. The reference strain *C. albicans* SC5314 was used as control and biofilm content is represented by the striped bar. Biofilm biomass production was quantified by measuring OD at 590 nm upon crystal violet release. The p-values for the groups for the fluconazole-resistant and caspofungin-resistant groups of isolates relative to the control are shown.

**Fig 9:**
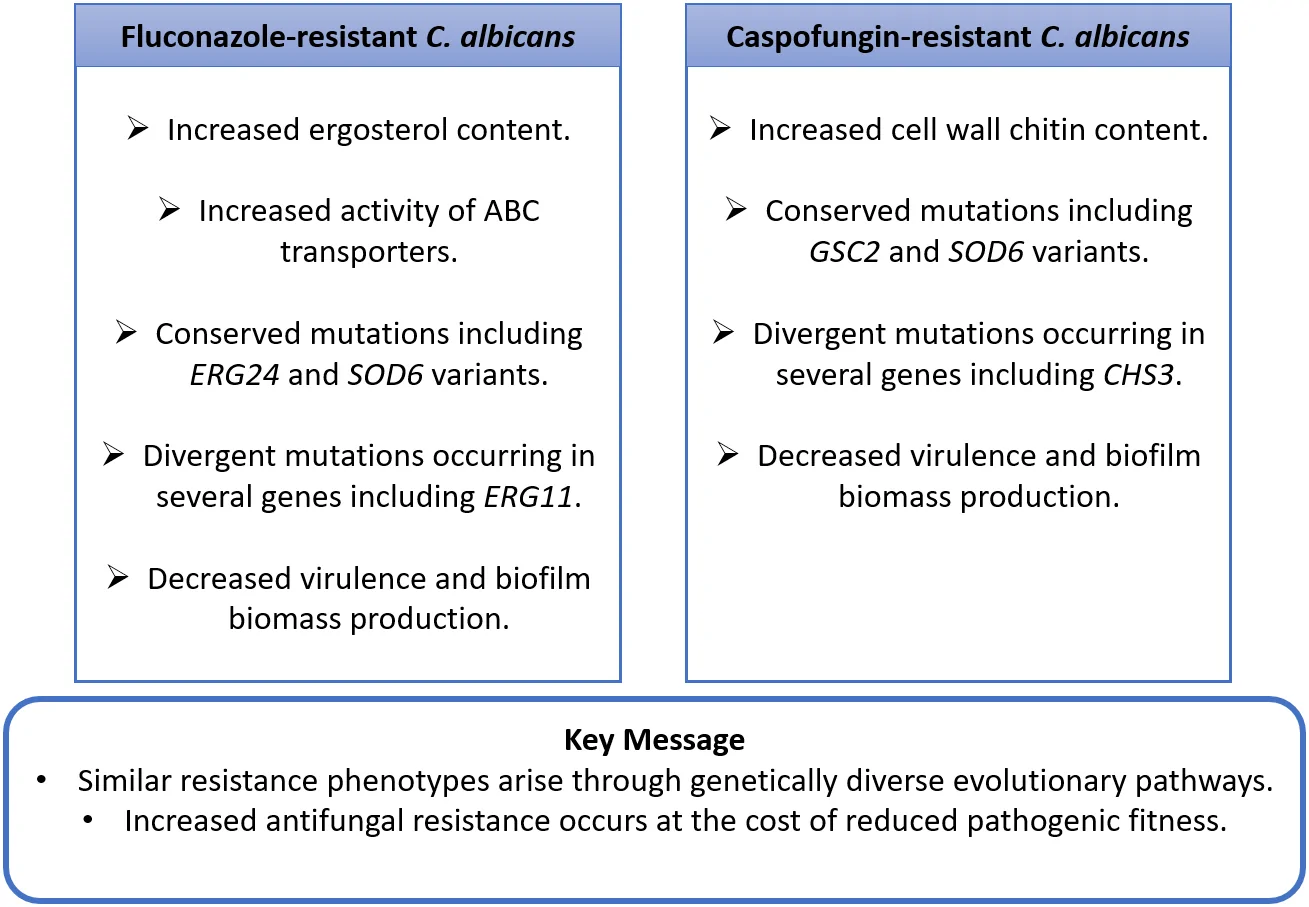
Summary of key findings and conclusions.

## Discussion

Over the past decade, hospital isolates of *C. albicans* have experienced increased antifungal resistance [4,20–23] but understanding how *C. albicans* acquires antifungal resistance remains a major challenge in fungal pathogenesis research [9,24]. The study of El Hachem et al. [19] induced an experimental evolution of resistance under antifungal selective pressures and generated fluconazole and caspofungin-resistant isolates exposed to final concentrations of 16 μg/mL fluconazole and 2 μg/mL caspofungin, respectively. Six randomly selected fluconazole-resistant (FL1 to FL6) and 6 other randomly selected caspofungin-resistant (CS1 to CS6) isolates of the highest resistance category were subjected to further analyses in the current study by integrating whole-genome sequencing with phenotypic assays linked to antifungal resistance and virulence. Our main aim was to determine whether isolates exposed to similar selective pressures converge on common resistance mechanisms or follow diverse evolutionary trajectories.

Whole-genome sequencing revealed extensive mutational heterogeneity across isolates, each accumulating a unique set of variants in comparison to the reference genome and none of the isolates shared an identical mutational profile (Tables 1 and 3). We focused on 34 genes (Fig 10) in fluconazole-resistant isolates and 22 genes carried mutations potentially linked to resistance and virulence (Table 1). In caspofungin-resistant isolates, 31 genes were examined and mutations were detected in 20 genes that may contribute to resistance and virulence mechanisms (Table 3). The genes analyzed were selected based on their established role in antifungal resistance and virulence according to the Candida Genome Database (CDG: https://www.candidagenome.org/).

**Fig 10:**
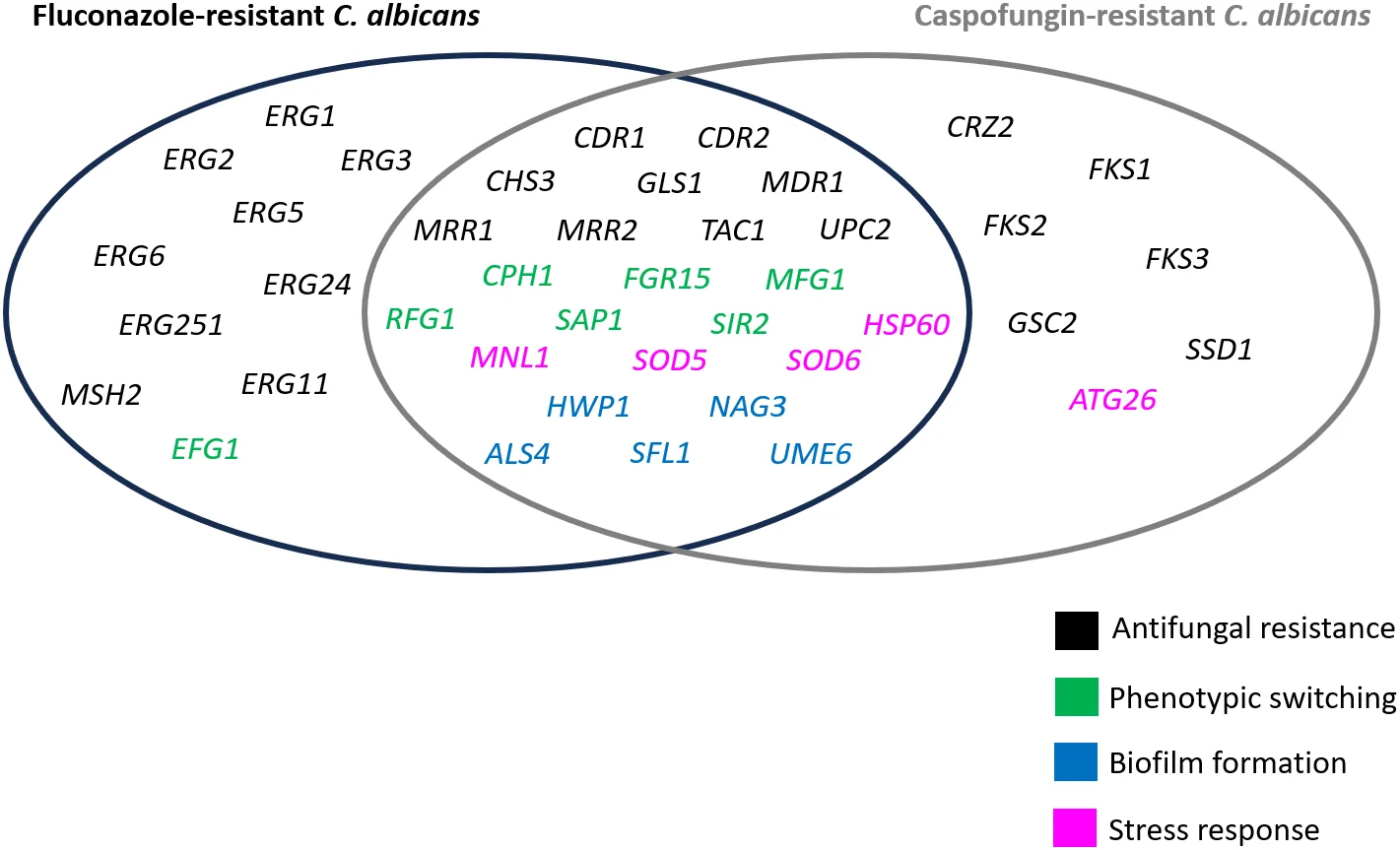
Targeted genes analyzed in fluconazole-resistant (FL1-FL6) and caspofungin-resistant (CS1-CS6) isolates.

Mash-derived ANI analyses revealed that all antifungal-resistant isolates exhibited a slightly lower genome-wide similarity to the reference strain *C. albicans* SC5314, indicating genomic divergence following experimental induction of antifungal resistance. Because Mash estimates ANI from shared *k*-mers rather than whole-genome alignments, it provides a descriptive measure of overall genomic relatedness that is useful for comparing closely related genomes and identifying clustering patterns rather than testing statistical significance. Similar applications of ANI analyses have been recently reported for *C. albicans*, where highly related isolates display very high ANI values while progressively divergent isolates separate into distinct genomic clusters, reflecting accumulated mutations [25]. Although all resistant isolates remained highly similar to one another, consistent with their origin from the same parental strain, both fluconazole-resistant and caspofungin-resistant isolates displayed measurable divergence from the reference genome (Fig 3). Within the fluconazole-resistant group of isolates, ANI values remained highly comparable among all 6 isolates (Fig 1), suggesting that independent adaptation occurred without substantial genome-wide divergence despite acquisition of distinct mutations. A similar pattern was observed for the caspofungin-resistant isolates, with the exception of CS6 which exhibited lower ANI values compared to other caspofungin-resistant isolates (Fig 2). Supported by the other genomic analysis in this manuscript, CS6 seemed to accumulate a greater number of sequence variants, suggesting that it followed a more divergent evolutionary trajectory while converging on the same resistant phenotype. The combination of high intra-group similarity and modest divergence from the parental strain supports the concept that resistance emerged through independent evolutionary events and that antifungal pressure limits adaptation to a specific set of solutions at the cellular level; such as increased ergosterol and chitin deposition, even if the adopted genetic routes are variable.

YMAP (Yeast Mapping Analysis Pipeline) profile analyses (S1 File) across both drug-evolved panels, showed differences from the *C. albicans* SC5314 parental profile that were driven primarily by copy number alterations and associated allele-ratio shifts. The fluconazole-resistant isolates span a spectrum from the near-euploid FL4 to extensive segmental copy number alterations. In the caspofungin-resistant isolates, genome-scale changes are similarly heterogeneous, ranging from largely diploid isolates with a single focal amplification to isolates with chromosome-scale copy gains and losses. Overall, YMAP supports that drug pressure frequently coincides with genome-structure remodeling dominated by copy number gains and losses, but the affected chromosomes, copy number variations, and amplitudes vary substantially among independently evolved isolates, arguing against a single convergent karyotypic signature for either drug in this set.

Ergosterol biosynthesis plays a key role in fluconazole resistance since upregulation of *ERG* genes or mutations in *ERG11* can partially restore ergosterol levels despite inhibition of lanosterol 14α-demethylase [13,26,27]. Fluconazole-resistant isolates showed strong increases (Fig 5) reflecting the occurrence of changes in the ergosterol biosynthesis pathway, a known target of azoles. Several isolates carried mutations in *ERG11* and *ERG4* as in a previous report linking sterol pathway remodeling to azole resistance [19]. For example, T128K harbored by Erg11 was detected in FL1, FL3, and FL6 (Table 1) as well as in fluconazole-resistant isolates from El Hachem et al. [19]. T128K was previously detected in fluconazole-resistant *C. albicans* [28]. Moreover, M157V harbored by Erg4 in two of our fluconazole-resistant isolates (Table 1) was also reported in El Hachem et al. [19]. In addition, R18G was detected in one of our fluconazole-resistant isolates. Although the latter mutations are novel, they might have led to *ERG4* overexpression which is an important factor linked to azole resistance [29]. Notably, the novel Q46H and G119S substitutions were recurrently detected in Erg24 for the majority of our fluconazole-resistant isolates and seem to be recurrent mutations involved in fluconazole resistance, probably altering the sterol biosynthesis enzyme composition (Table 2). Taken together these results support the idea that membrane composition changes represent a conserved axis of adaptation when subjected to antifungal pressure.

In addition to mutations and changes in gene expression of *ERG11* and other genes involved in the ergosterol biosynthesis pathway as well as alterations in sterol composition, azole resistance is also mediated by efflux pump upregulation [13]. The R6G assay specifically assesses the activity of ABC transporters and no other efflux pumps [30]. ABC transporters include Cdr1 and Cdr2 which are under the control of the transcriptional regulator Tac1 [13]. Increased efflux activity was observed in 5 out of 6 fluconazole-resistant isolates (Fig 7) but data was not shown in Table 1 as mutational variations were not recorded in *CDR1*, *CDR2*, and *TAC1*, suggesting that transcriptional changes are inducing increased efflux pump activity [31]. Increased activation of efflux pumps belonging to the major facilitator superfamily; mainly Mdr1, can also be involved in fluconazole resistance [32]. Four isolates harbored the I325V amino acid substitution in Mdr1 (Table 1), potentially increasing its specificity to azole transport. No mutations were detected in *MRR1*, the regulator of *MDR1* but this does not exclude gene expression changes in *MDR1* and *MRR1*.

Similar to ergosterol, chitin content also experienced an increase in all isolates (Fig 6). Chitin is a rigid cell wall polymer that can resist stress, including antifungal pressure. Its increased production has been previously shown to play a key role in caspofungin resistance [18,33]. In caspofungin-resistant isolates, increased chitin deposition can result from inhibition of β-1,3-glucan synthesis which triggers a compensatory reinforcement of the cell wall [18,33,34]. Although many mutations were detected in the chitin synthase gene *CHS3* across multiple isolates, none were conserved across caspofungin-resistant isolates (Table 3) which indicates that chitin accumulation might be achieved through diverse genetic routes rather than a single recurrent mutation. This finding is consistent with previous studies showing that increased chitin content is a compensatory response to echinocandin-induced cell wall stress and can result from activation of several cell wall stress response pathways such as the Ca^2+^-calcineurin, high-osmolarity glycerol, and protein kinase C cell wall integrity pathways [18,34–36]. All *CHS3* mutations detected in caspofungin-resistant isolates were also found in fluconazole-resistant isolates (Tables 1 and 3). These mutations likely contribute to increased chitin deposition since they occur in both resistant groups, suggesting enhanced Chs3 activity through gain-of-function effects. Although *CHS3* mutations seem to be associated with increased chitin production, the absence of recurrent mutations in caspofungin-resistant isolates was noted (Table 4). This mutational pattern suggests different Chs3 substitutions converge on the same phenotype of increased chitin production under caspofungin selective pressure. Caspofungin-resistant isolates also exhibited elevated ergosterol levels (Fig 5) and fluconazole-resistant isolates showed increased chitin deposition compared to the control (Fig 6) suggesting plasma membrane and cell wall remodeling may represent broader stress-adaptive phenotypes. Even though fluconazole mainly targets ergosterol biosynthesis, it can also lead to significant changes in the cell wall proteome and compromise cell wall integrity [37]. This points to evolutionary adaptation favoring a group of recurrent advantageous adaptive genetic changes. Furthermore, caspofungin-resistant isolates depend on increased chitin production for survival because caspofungin; like all echinocandins, can directly weaken the cell wall of *C. albicans* by damaging the β-1,3-glucan network. Under echinocandin selective pressure, any *CHS3* mutation that raises chitin levels even slightly, can favor survival [34,36]. As a result, diverse *CHS3* mutations are maintained, preventing the emergence of dominant recurrent mutations. This may support the detection of *CHS3* mutations in both resistance groups but recurrent mutations are mainly found in fluconazole-resistant isolates while caspofungin-resistant isolates exhibit mutational diversity (Tables 1–3). Notably, elevated levels of chitin deposition might be correlated to *CHS3* mutations since chitin increases reported by El Hachem et al. [19] were detected in absence of *CHS3* mutations and were moderate compared with the more pronounced increases observed in our current isolates.

Before acquiring resistance, *C. albicans* has to first activate stress response pathways that will eventually lead to cell wall and plasma membrane remodeling. In this study, both fluconazole and caspofungin-resistant isolates displayed almost the same accessory and recurrent mutations across the oxidative stress response genes *SOD5* and *SOD6* as well as *HSP60* and *MNL1* which are other stress response actors (Tables 1–4). Mutations in these genes likely reflected general drug-adaptation responses because exposure to both drugs selected similar stress-response mutations that are independent of antifungal-specific resistance mechanisms. These mutations might enhance tolerance to antifungal-induced stress that would favor resistance development [38,39].

In addition to all the above antifungal resistance mechanisms, increased biofilm formation could prevent antifungal compounds from reaching their respective targets in *C. albicans*, leading to antifungal tolerance and potentially resistance development [40,41]. However, all our isolates displayed reduced biofilm biomass compared to the reference strain (Fig 8) in the same manner as previous reports describing reduced biofilm formation in antifungal resistant *C. albicans*, particularly azole resistant isolates [19,42,43]. The observed decrease in biofilm formation in fluconazole and caspofungin-resistant isolates suggests that resistance evolution imposes fitness trade-offs that reduce virulence phenotypic characteristics [44]. Under antifungal stress, *C. albicans* activates survival and adaptation mechanisms such as ergosterol remodeling, efflux pump upregulation, increased chitin synthesis, and activation of stress response pathways. In *ALS4*, *HWP1*, *NAG3*, *SFL1*, and *UME6* which are the studied group of genes involved in biofilm formation, almost all mutations were common to the fluconazole and caspofungin-resistant isolates (Tables 1 and 3). However, caspofungin-resistant isolates did not carry recurrent mutations in genes implicated in biofilm formation unlike fluconazole-resistant isolates that harbored the recurrent changes A397V in Nag3, N257S in Ume6, as well as T75A and S373L in Sfl1 (Tables 2 and 4). The absence of recurrent mutations in biofilm-associated genes suggests that caspofungin-resistant isolates did not follow a uniform genetic pathway leading to biofilm loss. In contrast, fluconazole-resistant isolates accumulated more recurrent mutations in key biofilm regulators which indicates a more shared genetic basis for the observed biofilm reduction.

Antifungal resistance can impose fitness costs that reduce virulence, although this relationship is not universal and depends on the underlying resistance mechanism and associated genomic changes [45]. Consistent with this concept, the murine disseminated infection model revealed heterogeneous virulence among the resistant isolates (Fig 4), indicating that resistance acquisition did not uniformly alter pathogenicity. Most fluconazole-resistant isolates showed delayed mortality compared to the reference strain, while the caspofungin-resistant isolates generally retained virulence levels similar or higher compared to the reference strain. These observed phenotypic differences were consistent with the genomic analyses. Fluconazole-resistant isolates accumulated numerous mutations in genes involved in adhesion, filamentation, and host interaction including *ALS4*, *CPH1*, *EFG1*, *HWP1*, *SAP1*, and *SFL1* (Table 1), several of which regulate biofilm formation and hyphal development which are major determinants of *C. albicans* pathogenicity. Therefore, their reduced virulence likely reflects the cumulative fitness cost associated with multiple mutations affecting virulence-associated pathways, in agreement with the reduced biofilm formation observed in these isolates. In contrast, although the caspofungin-resistant isolates also accumulated numerous genomic changes including mutations affecting cell wall remodeling, most retained high virulence *in vivo* suggesting that the mechanisms conferring echinocandin resistance did not impair host adaptation. The greater genomic divergence observed in the ANI analysis of isolate CS6, did not associate with a markedly altered virulence, suggesting that overall genomic divergence is not necessarily predictive of pathogenicity. Altogether, these findings indicate that resistance evolved through distinct genomic trajectories that differentially affected virulence-associated pathways, supporting the concept that antifungal adaptation can preserve pathogenic potential despite extensive genomic remodeling. From a clinical perspective, this could be concerning because resistant isolates may remain highly virulent while simultaneously acquiring mutations that enhance survival under antifungal pressure.

One limitation of this study is that experimental evolution does not fully capture the complexity of clinical infection environments where many factors are at play such as the hosts immunity and niche-specific pressures among others. Another limitation includes the small sample size. Although our genomic analyses detected mutations associated with resistance phenotypes, functional validation was beyond the scope of this project, especially that the main purpose of the study is to compare genomic responses amongst isolates. Despite these limitations, our study supports a model in which antifungal resistance evolution in *C. albicans* seems to be characterized by sequence-level divergence coupled with pathway level convergence. Also, development of antifungal resistance seems to be supported by a subset of recurrent adaptive mutations while a broader set of accessory mutations promote evolutionary flexibility. As a future direction, transcriptomic analysis targeting candidate genes should be conducted to evaluate gene expression levels. Furthermore, the genomic and transcriptomic profiles of our isolates should be compared with those of publicly available fluconazole-resistant and caspofungin-resistant *C. albicans* isolates to place our findings in a broader context.

To sum up, this study demonstrated that different *C. albicans* isolates subjected to the same concentrations of fluconazole or caspofungin develop resistance through highly diverse mutational trajectories that converge on shared adaptive phenotypes including plasma membrane and cell wall remodeling, stress adaptation, and upregulation of efflux pumps (Fig 9). Altogether, our findings highlight the predictability of antifungal resistance at the pathway level but show diversity at the genetic level, emphasizing the importance of pathway-focused prophylactic and therapeutic strategies.

## Materials and methods

### *C. albicans* isolates

The reference strain *C. albicans* SC5314 (ATCC MYA-2876) was used as control. This same strain was incubated with fluconazole (Thermo Scientific, Waltham, MA, USA) or caspofungin (Sigma-Aldrich, St. Louis, MO, USA) at sequentially increasing concentrations at 2-week intervals over 3 months according to the protocol from El Hachem et al. [19]. The final drug concentrations to which the *C. albicans* reference strain was exposed were 16 μg/mL of fluconazole and 2 μg/mL of caspofungin. *C. albicans* grown in Roswell Park Memorial Institute (RPMI-1640) medium (Sigma-Aldrich, St. Louis, MO, USA) supplemented with each drug at the aforementioned highest concentrations were plated on potato dextrose agar (Conda Laboratories, Madrid, Spain) and incubated overnight at 35°C. Six random colonies of different sizes were isolated per condition and grown overnight in RPMI-1640 supplemented with the respective drug concentration before being stored in 15% glycerol at −80°C. FL1 to FL6 designate colonies of *C. albicans* exposed to a final concentration of 16 μg/mL of fluconazole and CS1 to CS6 those subjected to a final concentration of 2 μg/mL of caspofungin.

### Whole-genome sequencing and bioinformatic analyses

Genomic DNA was extracted using the ZR Fungal/Bacterial DNA MiniPrep kit (Zymo Research, Irvine, CA, USA) according to the manufacturers’ instructions. Whole-genome sequencing at 90x coverage was carried out by MicrobesNG (University of Birmingham, Birmingham, UK). DNA libraries were prepared using Nextera XT Library Preparation Kit (Illumina, San Diego, CA, USA) on a Hamilton Microlab STAR automated liquid handling system (Hamilton Bonaduz AG, Switzerland) and sequenced on an Illumina NovaSeq 6000 platform (Illumina, San Diego, CA, USA) using a 250 bp paired-end protocol. Raw sequence reads were adapter-trimmed and quality-filtered using Trimmomatic v0.30 [46], followed by genome assembly with SPAdes v3.7 [47] and genome annotation using Prokka v1.11 [48]. The whole-genome sequencing data generated in this study have been deposited in the NCBI GenBank under BioProject number PRJNA1427196 (https://submit.ncbi.nlm.nih.gov/subs/wgs_batch/SUB16037922/overview). Isolates FL1, FL2, FL3, FL4, FL5, and FL6 have the corresponding accession numbers respectively: SAMN56300551, SAMN56300552, SAMN56300553, SAMN56300554, SAMN56300555, and SAMN56300556. Isolates CS1, CS2, CS3, CS4, CS5, and CS6 have the corresponding accession numbers respectively: SAMN56300557, SAMN56300558, SAMN56300559, SAMN56300560, SAMN56300561, and SAMN56300562.

Genome-wide single nucleotide polymorphism (SNP) analysis was performed using the Snippy pipeline v4.5.0 [48] in which whole-genome sequences of *C. albicans* SC5314 genomes and the studied isolates were aligned against the reference strain *C. albicans* SC5314. SNPs were identified for each isolate and recurrent genome SNPs shared across all samples were extracted to generate a multiple sequence alignment. One file per isolate listing all the detected nucleotide variations compared to the reference genome was generated. Gene prediction was conducted using the BRAKER2 pipeline v2.1.6 in fungal mode which integrates GeneMark-ES v4.65 and AUGUSTUS v3.4.0 for fungal gene prediction, enabling the identification of gene models along with their corresponding coding sequences and mRNA features [49–53]. InterProScan v5.50-84.0 [54] was used on the Cluster of Orthologous Groups of proteins (COG) database to create the.xml files to incorporate in the functional annotation pipeline created by Funannotate v.1.8.7 [55]. The pipeline starts by running HMMscan implemented in the HMMER suite v3.3 against the Pfam database. Orthology-based functional classification was subsequently performed using eggNOG-mapper (emapper) v2.1.2 based on eggNOG orthology assignments [56,57]. Protein sequence similarity searches were conducted using DIAMOND BLASTp against the UniProt database v2021_02 and MEROPS protease database v12.0 [58]. The resulting annotations were consolidated using Gene2Product v1.69 and SignalP v5.0 was applied to predict secreted proteins [59]. The completeness and quality of genome assemblies and annotations were evaluated using BUSCO v5.2.2 [60]. In parallel, variant calling files generated independently by MicrobesNG following whole-genome sequencing were used for complementary analyses and detected mutations were visualized in Integrative Genomics Viewer (IGV) v2.16.2 [61]. Subsequent analyses focused on a targeted set of genes of known relevance in order to compare mutation patterns in fluconazole-resistant and caspofungin-resistant isolates. Analysis was focused on targeted genes involved in pathways that can possibly mediate antifungal resistance and adaptive stress responses (Fig 10). Gene names were retrieved from the Candida Genome Database (CGD: http://www.candidagenome.org/) [62].

### Pairwise genome similarity analysis

Pairwise genomic relatedness among isolates was assessed using Mash to estimate genome-wide distances [63]. Mash-derived distances were used to construct a pairwise genome similarity matrix, which was visualized as a heatmap using R statistical computing software. To facilitate interpretation of genomic similarity, Mash distances were converted to approximate average nucleotide identity (ANI) values. These ANI values represent approximate genome-wide similarity estimates based on shared *k*-mers rather than exact nucleotide identity. Consequently, lower ANI values indicate greater genomic divergence across the shared genomic fraction.

### Comparative chromosomal abnormality analyses

The Yeast Mapping Analysis Pipeline (YMAP) (http://lovelace.cs.umn.edu/Ymap/) was used to perform a comparative chromosomal abnormality analyses of all isolates versus the reference strain *C. albicans* SC5314 and the results are shown in S1 File.

### Murine model of systemic candidiasis

*In vivo* pathogenicity was assessed using a murine model of systemic *C. albicans* infection. The protocol was adapted from Khalaf et al., Fattouh et al., and El Hady et al. [64–66]. *C. albicans* isolates were inoculated into 50 mL of potato dextrose broth (PDB) (Conda Laboratories, Madrid, Spain) and incubated for 18 h at 30°C and 100 rpm. Each isolate was injected into one of the lateral caudal veins of 6 BALB/c female mice aged 6 weeks. For each injection, 10^7^ *C. albicans* were harvested by centrifugation for 5 min at 3000 rpm and resuspended in 0.2 mL of 1x phosphate buffered saline solution (PBS 1x). The number of moribund mice was evaluated on a daily basis. Mice were provided with food and water ad libitum. This experiment was conducted in compliance with the ethical standards of the Lebanese American University’s Institutional Animal Care and Use Committee and approved under protocol number LAU.ACUC.SAS.RK3.28/January/2022. Mice were housed under standard laboratory conditions. Accordingly, 6 mice were injected per strain (a total of 78 mice). Mice were monitored daily for a period of one month. Humane endpoints included refusal to eat, inability to move, or severe lethargy. As such, mice that appeared moribund were humanely euthanized by CO_2_ inhalation followed by cervical dislocation. All efforts were made to minimize animal suffering. In summary, out of 78 mice, 44 mice were found dead, and 34 were euthanized at humane endpoints. All staff involved in handling mice have CITI certificates on working with mice in research settings.

### Quantification of ergosterol content in *C. albicans*

*C. albicans* isolates were inoculated into 50 mL PDB at 35°C and 100 rpm for 24 h. Cultures were transferred to pre-weighed conical tubes and centrifuged for 5 min at 2700 rpm. The pellet weight was calculated before being suspended in 3 mL of 25% alcoholic potassium hydroxide by manual vortexing for 1 min. The suspensions were transferred to borosilicate glass screw cap tubes, incubated in an 85°C water bath for 1 h then cooled at room temperature for 15 min. Next, 1 mL of sterile water and 3 mL of *n*-heptane were added to the suspension and manually vortexed for 3 min. After vortexing, phase separation occurred within approximately 1 h of incubation at room temperature. The upper layers containing the extracted sterols were transferred to clean borosilicate glass screw cap tubes and stored for 24 h at −20°C. For quantification, 0.2 mL of the sterol-containing layer was mixed to 0.8 mL of pure ethanol and optical densities (OD) at 230 and 281.5 nm were measured using a Genesys 10S UV-Vis spectrophotometer (Thermo Fisher Scientific, Waltham, MA, USA). Ergosterol content was quantified as a percentage of the wet weight of the cell according to the “sterol quantification method (SQM)” protocol adapted from Arthington-Skaggs et al. [67] with some modifications. The mathematical formulas from Arthington-Skaggs et al. and Chedraoui et al. [67,68] were adopted. The experiment was performed in biological triplicates.

### Quantification of chitin content in *C. albicans*

*C. albicans* isolates were inoculated into 5 mL PDB and incubated overnight at 30°C and 80 rpm. The cell wall was extracted according to the “cell wall isolation” protocol of El Khoury et al. [69] and chitin was quantified based on the “determination of chitin content in cell walls” protocol adapted from Kapteyn et al. [70]Cultures were centrifuged for 5 min at 4000 rpm and the pellets were resuspended in 0.5 mL Tris buffer (5 mM, pH 7.8) and transferred to pre-weighed microcentrifuge tubes containing 3 pre-chilled undrilled glass beads of 3–5 mm in diameter (Himedia Laboratories, Mumbai, India). *C. albicans* cells in the pellet were disrupted by vortexing for 60 min using a multi-tube vortex mixer. The lysate was separated from the glass beads by being transferred to new pre-weighed microcentrifuge tubes. To ensure the complete recovery of lysates, the beads were washed with 0.5 mL of cold 1M NaCl, and the wash solution was combined with its respective sample. The samples were then centrifuged for 5 min at 3000 rpm. The pellet weights were determined before suspension in 0.5 mL protein extraction buffer (150 mM NaCl, 100 mM Na-EDTA, 50 mM Tris, 2% SDS, and 8 μL/mL β-mercaptoethanol, pH 7.8) per 100 mg of pellet wet weight. Suspensions were boiled at 100°C for 10 min, cooled for 5 min, and centrifuged for 5 min at 3000 rpm. The supernatant was discarded and the latter boiling, cooling, and centrifugation steps were repeated one more time. Pellets were then washed twice with 1 mL type II water to remove residual SDS and each step was followed by centrifugation for 5 min at 3000 rpm [69]. For extraction of cell wall chitin, 1 mL of 6N HCl was added to the pellets and boiled at 100°C for 10 min. After cooling, samples were centrifuged for 5 min at 3000 rpm and the pellets were re-suspended in 1 mL type II water. For quantification, 0.1 mL of sample was mixed with 0.1 mL of a 1.5 N Na_2_CO_3_ solution containing 4% acetylacetone. Mixtures were incubated at 100°C for 20 min then, cooled for 5 min at room temperature. Subsequently, 0.7 mL pure ethanol and 0.1 mL of a solution made of 1.6 g of p-dimethylbenzaldehyde dissolved in 30 mL concentrated HCl and 30 mL absolute ethanol were added. Samples were incubated at room temperature for 1h then OD at 520 nm was measured and chitin concentrations were calculated based on a glucosamine standard curve [70,71]. The experiment was performed in biological triplicates.

### Quantification of R6G efflux in *C. albicans*

The R6G assay was used to evaluate ATP-dependent efflux pump activity based on an adaptation of the protocol from Maesaki et al. [72]. This assay was only carried out on the fluconazole-resistant isolates because increased activity of ABC transporters is a well-established mechanism of azole resistance in *C. albicans*. R6G is a fluorescent substrate of ABC transporters, making it a suitable indicator of their efflux activity [72]. Only fluconazole-resistant *C. albicans* isolates were inoculated into 5 mL PDB in 50 mL conical tubes and incubated overnight at 32°C and 100 rpm. A total of 10^8^ cells were harvested and resuspended in 10 mL PDB then, centrifuged at 4°C for 7 min at 5000 rpm. The pellet was re-suspended in 10 mL PBS 1x and incubated at 32°C for 2h at 90 rpm to deplete intracellular ATP and equilibrate transport activity. To allow the uptake of the R6G dyes, 10 μL of 10 mM R6G was added and samples were incubated for an additional hour at 32°C and 90 rpm. Cells were pelleted by centrifugation at 4°C for 7 min at 5000 rpm and then, the pellet was washed twice with 10 mL PBS 1x under the same centrifugation conditions to remove residual extracellular dye. The final pellet was resuspended in 10 mL PBS 1x and incubated at 32°C and 90 rpm to initiate R6G efflux. At 15, 30, 45, and 60 min, 1.4 mL aliquots were collected and centrifuged at 9000 xg for 2 min and 1 mL of the supernatant was subjected to OD measurements at 527 nm [73]. This experiment was performed in biological triplicates and the mean OD per triplicate was calculated.

### Quantification of *C. albicans* biofilm biomass

*C. albicans* isolates were inoculated into 5 mL PDB and incubated overnight at 30°C and 80 rpm. In parallel, flat-bottom 96-well microtiter plates were pre-conditioned with 5% fetal bovine serum and incubated overnight at 4°C to enhance cell attachment and then, fetal bovine serum was washed out before adding a final concentration of 2 x 10^6^ cells/mL in PDB at a final volume of 0.2 mL in each well of the microtiter plate and incubated for 3 h at 37°C and 100 rpm. Following initial adhesion, the culture medium was removed and replaced by 0.2 mL fresh PDB and plates were incubated for 48 h at 37°C without shaking to allow biofilm formation. To quantify the biofilm biomass, PDB was removed and biofilms were fixed with 0.2 mL of 99% methanol for 15 min at room temperature. Methanol was discarded and plates were left to air-dry for 20 min. Biofilms were stained with 0.2 mL of 0.02% crystal violet for 20 min then, washed five times with type II water to remove excess stain. After 15 min of air-drying, crystal violet was released by adding 33% acetic acid to the wells and OD was measured at 590 nm using a Multiskan FC Microplate Photometer (Thermo Fisher Scientific, Rockford, IL, USA). This protocol was adapted from Fattouh et al. [65,74]. The experiment was performed in biological triplicates and each biological replicate was analyzed in three technical replicates from which the mean OD was calculated.

### Statistical analyses and data visualization

Statistical analyses and data presentation were carried out in GraphPad Prism v10.6.1. For ergosterol, chitin, and biofilm biomass quantification assays, Kruskal-Wallis test followed by Dunn’s multiple comparisons test was used to compare the fluconazole or caspofungin-resistant group of isolates to the control. For the quantification of R6G efflux, each fluconazole-resistant isolate was compared to the control using an ordinary two-way ANOVA with main effects only. For all statistical tests, p-values below 0.05 were considered significant.

## Supporting information

S1 File Summarized YMAP interpretation for fluconazole-resistant (FL1-FL6) and caspofungin-resistant (CS1-CS6) isolates compared to the reference strain *C. albicans* SC5314 (control). (PDF)

## References

[1] Denning DW. Global incidence and mortality of severe fungal disease. Lancet Infect Dis. 2024;24(7):e428–38. doi: 10.1016/S1473-3099(23)00692-8 38224705

[2] Turner SA, Butler G. The Candida pathogenic species complex. Cold Spring Harb Perspect Med. 2014;4(9):a019778. doi: 10.1101/cshperspect.a019778 25183855 PMC4143104

[3] Lamoth F, Lockhart SR, Berkow EL, Calandra T. Changes in the epidemiological landscape of invasive candidiasis. J Antimicrob Chemother. 2018;73(suppl_1):i4–13. doi: 10.1093/jac/dkx444 29304207 PMC11931512

[4] Parambath S, Dao A, Kim HY, Zawahir S, Izquierdo A-A, Tacconelli E, et al. Candida albicans-A systematic review to inform the World Health Organization Fungal Priority Pathogens List. Med Mycol. 2024;62(6):myae045. doi: 10.1093/mmy/myae045 38935906 PMC11210619

[5] Kmeid J, Jabbour J-F, Kanj SS. Epidemiology and burden of invasive fungal infections in the countries of the Arab League. J Infect Public Health. 2020;13(12):2080–6. doi: 10.1016/j.jiph.2019.05.007 31248814

[6] Ghaddar N, Anastasiadis E, Halimeh R, Ghaddar A, Dhar R, AlFouzan W, et al. Prevalence and antifungal susceptibility of Candida albicans causing vaginal discharge among pregnant women in Lebanon. BMC Infect Dis. 2020;20(1):32. doi: 10.1186/s12879-019-4736-2 31931738 PMC6958632

[7] El Zakhem A, Mahmoud O, Zakhour J, Nahhal SB, El Ghawi N, Omran N, et al. The impact of COVID-19 on the epidemiology and outcomes of candidemia: A retrospective study from a Tertiary Care Center in Lebanon. J Fungi (Basel). 2023;9(7):769. doi: 10.3390/jof9070769 37504757 PMC10381108

[8] Pappas PG, Kauffman CA, Andes DR, Clancy CJ, Marr KA, Ostrosky-Zeichner L, et al. Clinical practice guideline for the management of candidiasis: 2016 update by the Infectious Diseases Society of America. Clin Infect Dis. 2015;62:e1-50. doi: 10.1093/cid/civ933 PMC4725385 26679628

[9] Lee Y, Puumala E, Robbins N, Cowen LE. Antifungal drug resistance: Molecular mechanisms in Candida albicans and beyond. Chem Rev. 2021;121(6):3390–411. doi: 10.1021/acs.chemrev.0c00199 32441527 PMC8519031

[10] Noble SM, Gianetti BA, Witchley JN. Candida albicans cell-type switching and functional plasticity in the mammalian host. Nat Rev Microbiol. 2017;15(2):96–108. doi: 10.1038/nrmicro.2016.157 27867199 PMC5957277

[11] d’Enfert C, Kaune A-K, Alaban L-R, Chakraborty S, Cole N, Delavy M, et al. The impact of the Fungus-Host-Microbiota interplay upon Candida albicans infections: Current knowledge and new perspectives. FEMS Microbiol Rev. 2021;45(3):fuaa060. doi: 10.1093/femsre/fuaa060 33232448 PMC8100220

[12] Lopes JP, Lionakis MS. Pathogenesis and virulence of Candida albicans. Virulence. 2022;13(1):89–121. doi: 10.1080/21505594.2021.2019950 34964702 PMC9728475

[13] Whaley SG, Berkow EL, Rybak JM, Nishimoto AT, Barker KS, Rogers PD. Azole antifungal resistance in Candida albicans and emerging non-albicans Candida species. Front Microbiol. 2017;7:2173. doi: 10.3389/fmicb.2016.02173 28127295 PMC5226953

[14] Nishimoto AT, Sharma C, Rogers PD. Molecular and genetic basis of azole antifungal resistance in the opportunistic pathogenic fungus Candida albicans. J Antimicrob Chemother. 2020;75(2):257–70. doi: 10.1093/jac/dkz400 31603213 PMC8204710

[15] Arendrup MC, Perlin DS. Echinocandin resistance: An emerging clinical problem? Curr Opin Infect Dis. 2014;27(6):484–92. doi: 10.1097/QCO.0000000000000111 25304391 PMC4221099

[16] Shields RK, Nguyen MH, Clancy CJ. Clinical perspectives on echinocandin resistance among Candida species. Curr Opin Infect Dis. 2015;28(6):514–22. doi: 10.1097/QCO.0000000000000215 26524326 PMC4632243

[17] Perlin DS. Mechanisms of echinocandin antifungal drug resistance. Ann N Y Acad Sci. 2015;1354(1):1–11. doi: 10.1111/nyas.12831 26190298 PMC4626328

[18] Walker LA, Munro CA, de Bruijn I, Lenardon MD, McKinnon A, Gow NAR. Stimulation of chitin synthesis rescues Candida albicans from echinocandins. PLoS Pathog. 2008;4(4):e1000040. doi: 10.1371/journal.ppat.1000040 18389063 PMC2271054

[19] El Hachem S, Fattouh N, Chedraoui C, Finianos M, Bitar I, Khalaf RA. Sequential induction of drug resistance and characterization of an initial Candida albicans drug-sensitive isolate. J Fungi (Basel). 2024;10(5):347. doi: 10.3390/jof10050347 38786702 PMC11122215

[20] Kilbas I, Kahraman Kilbas EP, Horhat FG, Ciftci IH. Twenty-year course of antifungal resistance in Candida albicans in Türkiye: A systematic review and meta-analysis. J Fungi (Basel). 2025;11(8):603. doi: 10.3390/jof11080603 40863554 PMC12387985

[21] Nguyen MD, Ren P. Trends in antifungal resistance among Candida species: An eight-year retrospective study in the Galveston-Houston Gulf Coast Region. J Fungi (Basel). 2025;11(3):232. doi: 10.3390/jof11030232 40137269 PMC11943608

[22] Rabault C, Shuping L, Mpembe R, Quan V, Lanternier F, Lortholary O, et al. Recent systemic antifungal exposure and nonsusceptible Candida in hospitalized patients, South Africa, 2012-2017. Emerg Infect Dis. 2025;31(10):1901–11. doi: 10.3201/eid3110.250359 41017033 PMC12483017

[23] Kim K-Y, Kim G-Y, Kim JK. Trends in Candida albicans bloodstream infections and antifungal resistance in a Tertiary Care Hospital in South Korea (2013-2023). Mycobiology. 2025;53(2):236–42. doi: 10.1080/12298093.2025.2461336 40098941 PMC11912239

[24] Lee Y, Robbins N, Cowen LE. Molecular mechanisms governing antifungal drug resistance. NPJ Antimicrob Resist. 2023;1(1):5. doi: 10.1038/s44259-023-00007-2 38686214 PMC11057204

[25] Tsai M-H, Hsieh C-H, Tsai I-A, Chang C-M, Chen T-W, Hsu J-F, et al. Genetic variation and mutational determinants of azole resistance in Candida albicans strains of oropharyngeal colonization in HIV patients and bloodstream infections. J Biomed Sci. 2026;33(1):20. doi: 10.1186/s12929-026-01231-4 41724950 PMC12925357

[26] Berkow EL, Lockhart SR. Fluconazole resistance in Candida species: A current perspective. Infect Drug Resist. 2017;10:237–45. doi: 10.2147/IDR.S118892 28814889 PMC5546770

[27] Eliaš D, Tóth Hervay N, Gbelská Y. Ergosterol biosynthesis and regulation impact the antifungal resistance and virulence of Candida spp. Stresses. 2024;4(4):641–62. doi: 10.3390/stresses4040041

[28] Peron IH, Reichert-Lima F, Busso-Lopes AF, Nagasako CK, Lyra L, Moretti ML, et al. Resistance surveillance in Candida albicans: A five-year antifungal susceptibility evaluation in a Brazilian University Hospital. PLoS One. 2016;11(7):e0158126. doi: 10.1371/journal.pone.0158126 27414653 PMC4945058

[29] Feng W, Yang J, Xi Z, Qiao Z, Lv Y, Wang Y, et al. Mutations and/or overexpressions of ERG4 and ERG11 genes in clinical azoles-resistant isolates of Candida albicans. Microb Drug Resist. 2017;23(5):563–70. doi: 10.1089/mdr.2016.0095 27976986

[30] Bhattacharya S, Sobel JD, White TC. A combination fluorescence assay demonstrates increased efflux pump activity as a resistance mechanism in azole-resistant vaginal Candida albicans isolates. Antimicrob Agents Chemother. 2016;60(10):5858–66. doi: 10.1128/AAC.01252-16 27431223 PMC5038269

[31] Coste AT, Karababa M, Ischer F, Bille J, Sanglard D. TAC1, transcriptional activator of CDR genes, is a new transcription factor involved in the regulation of Candida albicans ABC transporters CDR1 and CDR2. Eukaryot Cell. 2004;3(6):1639–52. doi: 10.1128/EC.3.6.1639-1652.2004 15590837 PMC539021

[32] Rogers TR, Verweij PE, Castanheira M, Dannaoui E, White PL, Arendrup MC, et al. Molecular mechanisms of acquired antifungal drug resistance in principal fungal pathogens and EUCAST guidance for their laboratory detection and clinical implications. J Antimicrob Chemother. 2022;77(8):2053–73. doi: 10.1093/jac/dkac161 35703391 PMC9333407

[33] Lee KK, Maccallum DM, Jacobsen MD, Walker LA, Odds FC, Gow NAR, et al. Elevated cell wall chitin in Candida albicans confers echinocandin resistance in vivo. Antimicrob Agents Chemother. 2012;56(1):208–17. doi: 10.1128/AAC.00683-11 21986821 PMC3256049

[34] Walker LA, Gow NAR, Munro CA. Elevated chitin content reduces the susceptibility of Candida species to caspofungin. Antimicrob Agents Chemother. 2013;57(1):146–54. doi: 10.1128/AAC.01486-12 23089748 PMC3535899

[35] Lenardon MD, Munro CA, Gow NAR. Chitin synthesis and fungal pathogenesis. Curr Opin Microbiol. 2010;13(4):416–23. doi: 10.1016/j.mib.2010.05.002 20561815 PMC2923753

[36] Munro CA, Selvaggini S, de Bruijn I, Walker L, Lenardon MD, Gerssen B, et al. The PKC, HOG and Ca2+ signalling pathways co-ordinately regulate chitin synthesis in Candida albicans. Mol Microbiol. 2007;63(5):1399–413. doi: 10.1111/j.1365-2958.2007.05588.x 17302816 PMC2649417

[37] Sorgo AG, Heilmann CJ, Dekker HL, Bekker M, Brul S, de Koster CG, et al. Effects of fluconazole on the secretome, the wall proteome, and wall integrity of the clinical fungus Candida albicans. Eukaryot Cell. 2011;10(8):1071–81. doi: 10.1128/EC.05011-11 21622905 PMC3165447

[38] Robbins N, Caplan T, Cowen LE. Molecular evolution of antifungal drug resistance. Annu Rev Microbiol. 2026;27:12. doi: 10.1146/annurev-micro-030117 28886681

[39] Hokken MWJ, Zwaan BJ, Melchers WJG, Verweij PE. Facilitators of adaptation and antifungal resistance mechanisms in clinically relevant fungi. Fungal Genet Biol. 2019;132:103254. doi: 10.1016/j.fgb.2019.103254 31326470

[40] Cavalheiro M, Teixeira MC. Candida biofilms: Threats, challenges, and promising strategies. Front Med (Lausanne). 2018;5:28. doi: 10.3389/fmed.2018.00028 29487851 PMC5816785

[41] Kaur J, Nobile CJ. Antifungal drug-resistance mechanisms in Candida biofilms. Curr Opin Microbiol. 2023;71:102237. doi: 10.1016/j.mib.2022.102237 36436326 PMC11569868

[42] Panariello BHD, Klein MI, Mima EGDO, Pavarina AC. Fluconazole impacts the extracellular matrix of fluconazole-susceptible and -resistant Candida albicans and Candida glabrata biofilms. J Oral Microbiol. 2018;10(1):1476644. doi: 10.1080/20002297.2018.1476644 29887974 PMC5990947

[43] Bruzual I, Riggle P, Hadley S, Kumamoto CA. Biofilm formation by fluconazole-resistant Candida albicans strains is inhibited by fluconazole. J Antimicrob Chemother. 2007;59(3):441–50. doi: 10.1093/jac/dkl521 17261564

[44] Berman J, Krysan DJ. Drug resistance and tolerance in fungi. Nat Rev Microbiol. 2020;18(6):319–31. doi: 10.1038/s41579-019-0322-2 32047294 PMC7231573

[45] Cowen LE, Sanglard D, Howard SJ, Rogers PD, Perlin DS. Mechanisms of antifungal drug resistance. Cold Spring Harb Perspect Med. 2014;5(7):a019752. doi: 10.1101/cshperspect.a019752 25384768 PMC4484955

[46] Bolger AM, Lohse M, Usadel B. Trimmomatic: A flexible trimmer for Illumina sequence data. Bioinformatics. 2014;30(15):2114–20. doi: 10.1093/bioinformatics/btu170 24695404 PMC4103590

[47] Bankevich A, Nurk S, Antipov D, Gurevich AA, Dvorkin M, Kulikov AS, et al. SPAdes: A new genome assembly algorithm and its applications to single-cell sequencing. J Comput Biol. 2012;19(5):455–77. doi: 10.1089/cmb.2012.0021 22506599 PMC3342519

[48] Seemann T. Snippy: Fast bacterial variant calling from NGS reads. n .d.

[49] Stanke M, Keller O, Gunduz I, Hayes A, Waack S, Morgenstern B. AUGUSTUS: A b initio prediction of alternative transcripts. Nucleic Acids Res. 2006;34. doi: 10.1093/nar/gkl200 PMC1538822 16845043

[50] Ter-Hovhannisyan V, Lomsadze A, Chernoff YO, Borodovsky M. Gene prediction in novel fungal genomes using an ab initio algorithm with unsupervised training. Genome Res. 2008;18(12):1979–90. doi: 10.1101/gr.081612.108 18757608 PMC2593577

[51] Hoff KJ, Lange S, Lomsadze A, Borodovsky M, Stanke M. BRAKER1: Unsupervised RNA-Seq-based genome annotation with GeneMark-ET and AUGUSTUS. Bioinformatics. 2016;32(5):767–9. doi: 10.1093/bioinformatics/btv661 26559507 PMC6078167

[52] Brůna T, Hoff KJ, Lomsadze A, Stanke M, Borodovsky M. BRAKER2: Automatic eukaryotic genome annotation with GeneMark-EP+ and AUGUSTUS supported by a protein database. NAR Genom Bioinform. 2021;3(1):lqaa108. doi: 10.1093/nargab/lqaa108 33575650 PMC7787252

[53] Hoff K, Lomsadze A, Borodovsky M, Stanke M. Whole-genome annotation with BRAKER. Gene prediction: Methods and protocols. New York: Springer New York; 2019. p. 65–95. 10.1007/978-1-4939-9173-0_5 PMC6635606 31020555

[54] Jones P, Binns D, Chang H-Y, Fraser M, Li W, McAnulla C, et al. InterProScan 5: Genome-scale protein function classification. Bioinformatics. 2014;30(9):1236–40. doi: 10.1093/bioinformatics/btu031 24451626 PMC3998142

[55] Blachowicz A, Chiang AJ, Elsaesser A, Kalkum M, Ehrenfreund P, Stajich JE, et al. Proteomic and metabolomic characteristics of extremophilic fungi under simulated mars conditions. Front Microbiol. 2019;10:1013. doi: 10.3389/fmicb.2019.01013 31156574 PMC6529585

[56] Huerta-Cepas J, Forslund K, Coelho LP, Szklarczyk D, Jensen LJ, von Mering C, et al. Fast genome-wide functional annotation through orthology assignment by eggNOG-mapper. Mol Biol Evol. 2017;34(8):2115–22. doi: 10.1093/molbev/msx148 28460117 PMC5850834

[57] Huerta-Cepas J, Szklarczyk D, Heller D, Hernández-Plaza A, Forslund SK, Cook H, et al. eggNOG 5.0: A hierarchical, functionally and phylogenetically annotated orthology resource based on 5090 organisms and 2502 viruses. Nucleic Acids Res. 2019;47(D1):D309–14. doi: 10.1093/nar/gky1085 30418610 PMC6324079

[58] Buchfink B, Huson DH, Xie C. MetaScope - Fast and accurate identification of microbes in metagenomic sequencing data. 2015.

[59] Almagro Armenteros JJ, Tsirigos KD, Sønderby CK, Petersen TN, Winther O, Brunak S, et al. SignalP 5.0 improves signal peptide predictions using deep neural networks. Nat Biotechnol. 2019;37(4):420–3. doi: 10.1038/s41587-019-0036-z 30778233

[60] Manni M, Berkeley MR, Seppey M, Zdobnov EM. BUSCO: Assessing genomic data quality and beyond. Curr Protoc. 2021;1(12):e323. doi: 10.1002/cpz1.323 34936221

[61] Robinson JT, Thorvaldsdóttir H, Winckler W, Guttman M, Lander ES, Getz G, et al. Integrative genomics viewer. Nat Biotechnol. 2011;29(1):24–6. doi: 10.1038/nbt.1754 21221095 PMC3346182

[62] Skrzypek MS, Binkley J, Binkley G, Miyasato SR, Simison M, Sherlock G. The Candida Genome Database (CGD): Incorporation of Assembly 22, systematic identifiers and visualization of high throughput sequencing data. Nucleic Acids Res. 2017;45(D1):D592–6. doi: 10.1093/nar/gkw924 27738138 PMC5210628

[63] Ondov BD, Treangen TJ, Melsted P, Mallonee AB, Bergman NH, Koren S, et al. Mash: Fast genome and metagenome distance estimation using MinHash. Genome Biol. 2016;17(1):132. doi: 10.1186/s13059-016-0997-x 27323842 PMC4915045

[64] Khalaf RA, Fattouh N, Medvecky M, Hrabak J. Whole genome sequencing of a clinical drug resistant Candida albicans isolate reveals known and novel mutations in genes involved in resistance acquisition mechanisms. J Med Microbiol. 2021;70(4):001351. doi: 10.1099/jmm.0.001351 33909551 PMC8289213

[65] Fattouh N, Hdayed D, Geukgeuzian G, Tokajian S, Khalaf RA. Molecular mechanism of fluconazole resistance and pathogenicity attributes of Lebanese Candida albicans hospital isolates. Fungal Genet Biol. 2021;153:103575. doi: 10.1016/j.fgb.2021.103575 34033880

[66] El Hady R, Fattouh N, Finianos M, Bitar I, Fakih T, Husni R, et al. Phenotypic and genotypic characterization of Candida parapsilosis complex isolates from a Lebanese hospital. Sci Rep. 2025;15(1):4853. doi: 10.1038/s41598-024-84535-5 39924528 PMC11808099

[67] Arthington-Skaggs BA, Jradi H, Desai T, Morrison CJ. Quantitation of ergosterol content: Novel method for determination of fluconazole susceptibility of Candida albicans. J Clin Microbiol. 1999;37(10):3332–7. doi: 10.1128/JCM.37.10.3332-3337.1999 10488201 PMC85559

[68] Chedraoui C, Fattouh N, El Hachem S, Sourenian T, El Bitar L, El Moheb L, et al. Integrated genomic and phenotypic analyses reveal convergent resistance patterns in clinical Candida tropicalis isolates. Mycoses. 2026;69(5):e70181. doi: 10.1111/myc.70181 42037551 PMC13112332

[69] El Khoury P, Awad A, Wex B, Khalaf RA. Proteomic analysis of a Candida albicans pir32 null strain reveals proteins involved in adhesion, filamentation and virulence. PLoS One. 2018;13(3):e0194403. doi: 10.1371/journal.pone.0194403 29554112 PMC5858828

[70] Kapteyn JC, Hoyer LL, Hecht JE, Müller WH, Andel A, Verkleij AJ, et al. The cell wall architecture of Candida albicans wild-type cells and cell wall-defective mutants. Mol Microbiol. 2000;35(3):601–11. doi: 10.1046/j.1365-2958.2000.01729.x 10672182

[71] Fattouh N, Khalaf RA, Husni R. Candida glabrata hospital isolate from Lebanon reveals micafungin resistance associated with increased chitin and resistance to a cell-surface-disrupting agent. J Glob Antimicrob Resist. 2024;37:62–8. doi: 10.1016/j.jgar.2024.02.012 38408565

[72] Maesaki S, Marichal P, Vanden Bossche H, Sanglard D, Kohno S. Rhodamine 6G efflux for the detection of CDR1-overexpressing azole-resistant Candida albicans strains. J Antimicrob Chemother. 1999;44(1):27–31. doi: 10.1093/jac/44.1.27 10459807

[73] Chedraoui C, Fattouh N, El Hachem S, Younes M, Khalaf RA. Induction of antifungal tolerance reveals genetic and phenotypic changes in Candida glabrata. J Fungi (Basel). 2025;11(4):284. doi: 10.3390/jof11040284 40278105 PMC12028409

[74] Fattouh N, Husni R, Finianos M, Bitar I, Khalaf RA. Adhesive and biofilm-forming Candida glabrata Lebanese hospital isolates harbour mutations in subtelomeric silencers and adhesins. Mycoses. 2024;67(6):e13750. doi: 10.1111/myc.13750 38813959

