# Supplementary material for: Diverse genetic pathways drive convergent fluconazole and caspofungin resistance phenotypes in Candida albicans
Source: PLoS One. 2026 Oct 9;21(10):e0360234. doi: 10.1371/journal.pone.0360234 (PMC13652276; doi:10.1371/journal.pone.0360234)

S1 File. Summarized YMAP interpretation for fluconazole-resistant (FL1-FL6) and caspofungin-resistant (CS1-CS6) isolates compared to the reference strain *C. albicans* SC5314 (control). Chromosomes 1 to 7 and chromosome R are shown. For each chromosome, the left panel displays the B-allele frequency (BAF) histogram, indicating the distribution of homozygous (a and b) and heterozygous (ab) single nucleotide polymorphisms (SNPs). The central panel presents chromosome coordinates in Mb (x-axis) with sequencing read depths used to estimate chromosome copy number (y-axis). The black vertical bars represent normalized read depths across genomic windows. The chromosome ideograms depict gene distribution and centromere locations. The red tick marks highlight chromosomal landmarks represented in the right panel. The right panel summarizes the estimated copy number across each chromosomal segment. A copy number value > 2.5 was interpreted as a copy number gain, whereas a value < 1.5 was considered a copy number loss. The truncation of certain chromosome termini reflects the limited mappability and incomplete assembly of highly repetitive telomeric and subtelomeric regions in the reference genome.

(A) The diploid reference strain *C. albicans* SC5314 (control).

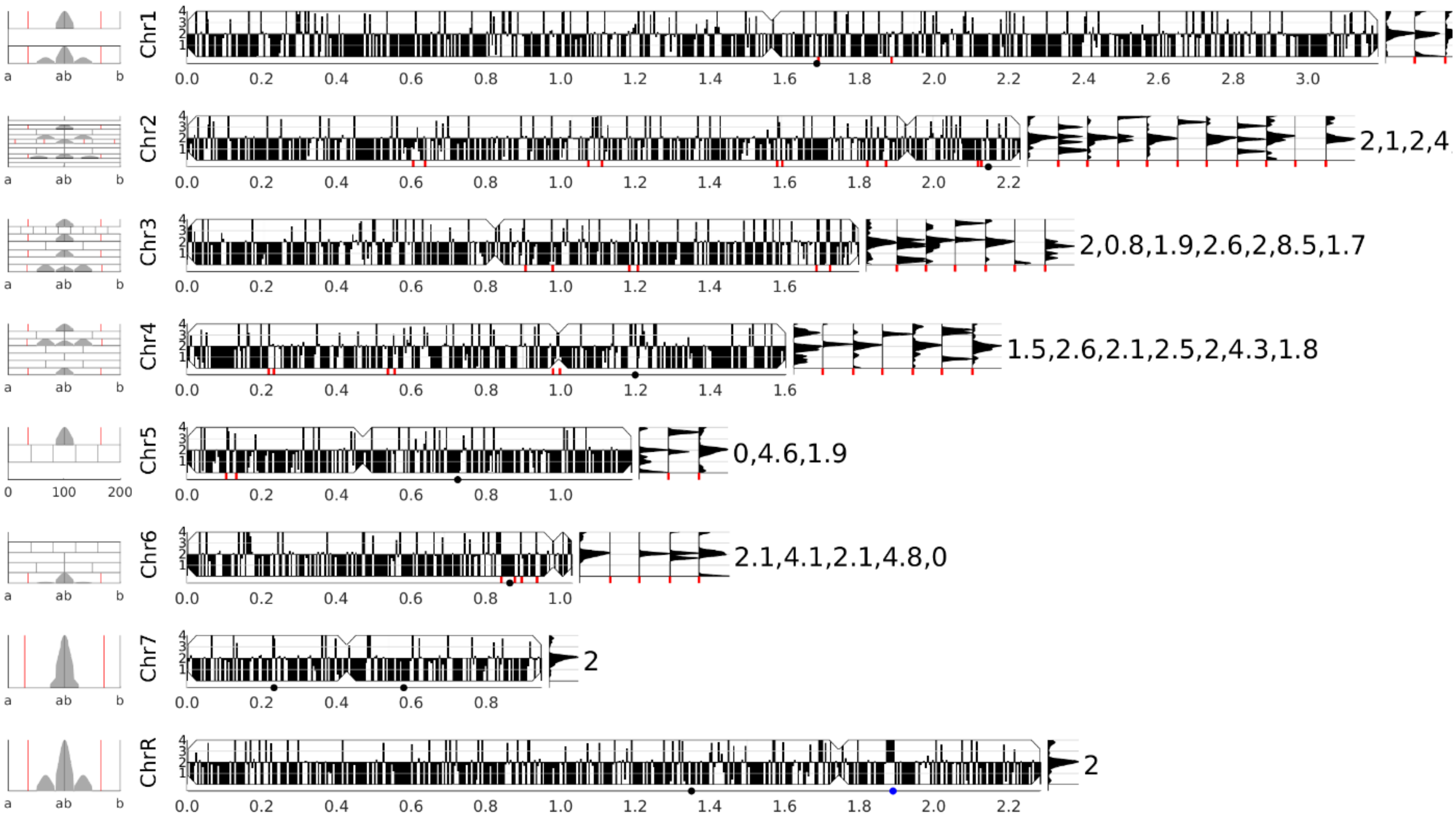

(B) Fluconazole-resistant isolate FL1: Compared to the control, FL1 maintained a largely stable diploid genome with preserved heterozygosity across all chromosomes. A localized copy number gain was observed on chromosome 4 and a loss was observed on chromosome 6.

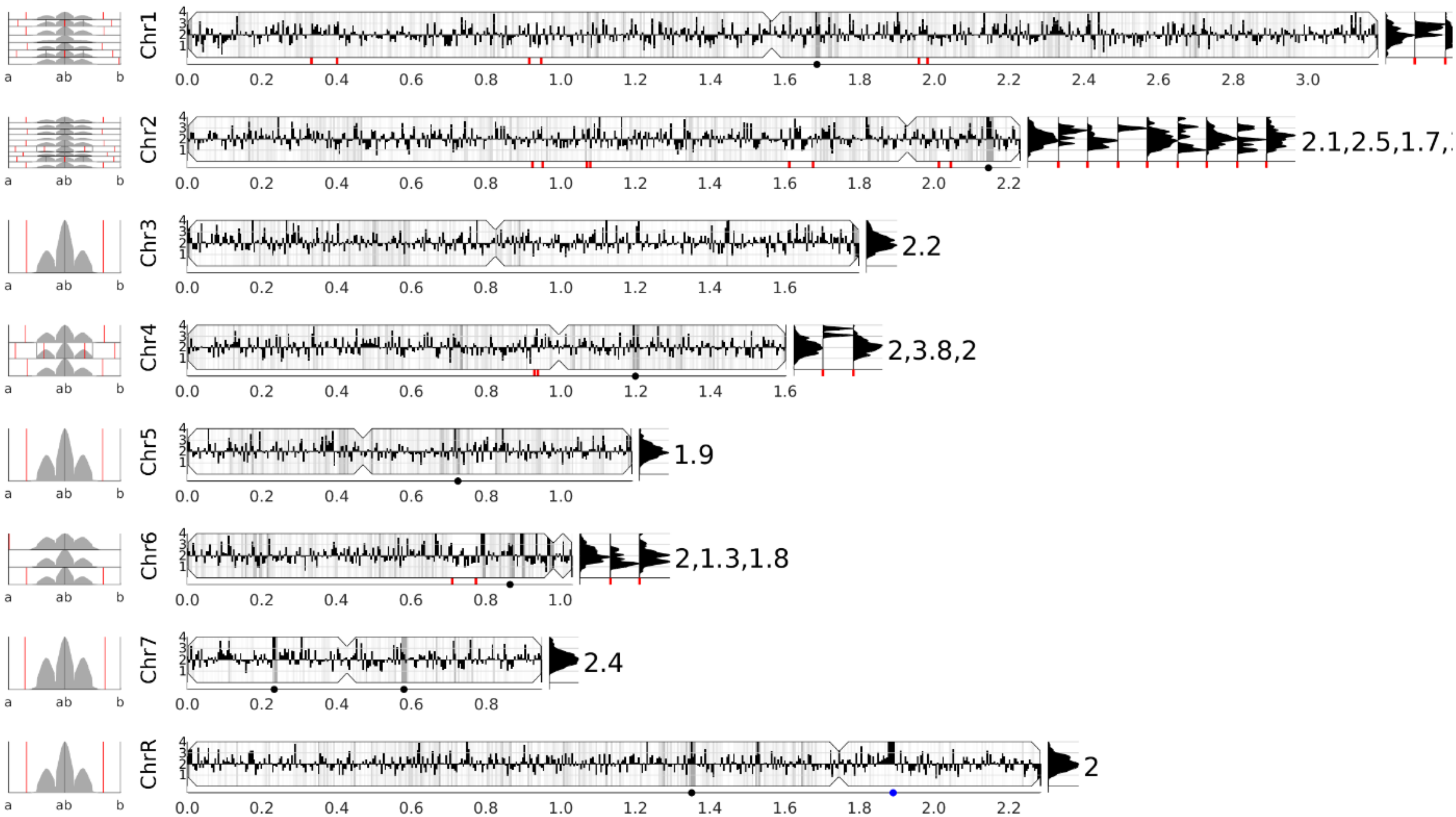

(C) Fluconazole-resistant isolate FL2: Compared to the control, FL2 maintained a largely stable diploid genome with preserved heterozygosity across all chromosomes. Localized copy number gains and a loss were observed on chromosome 6.

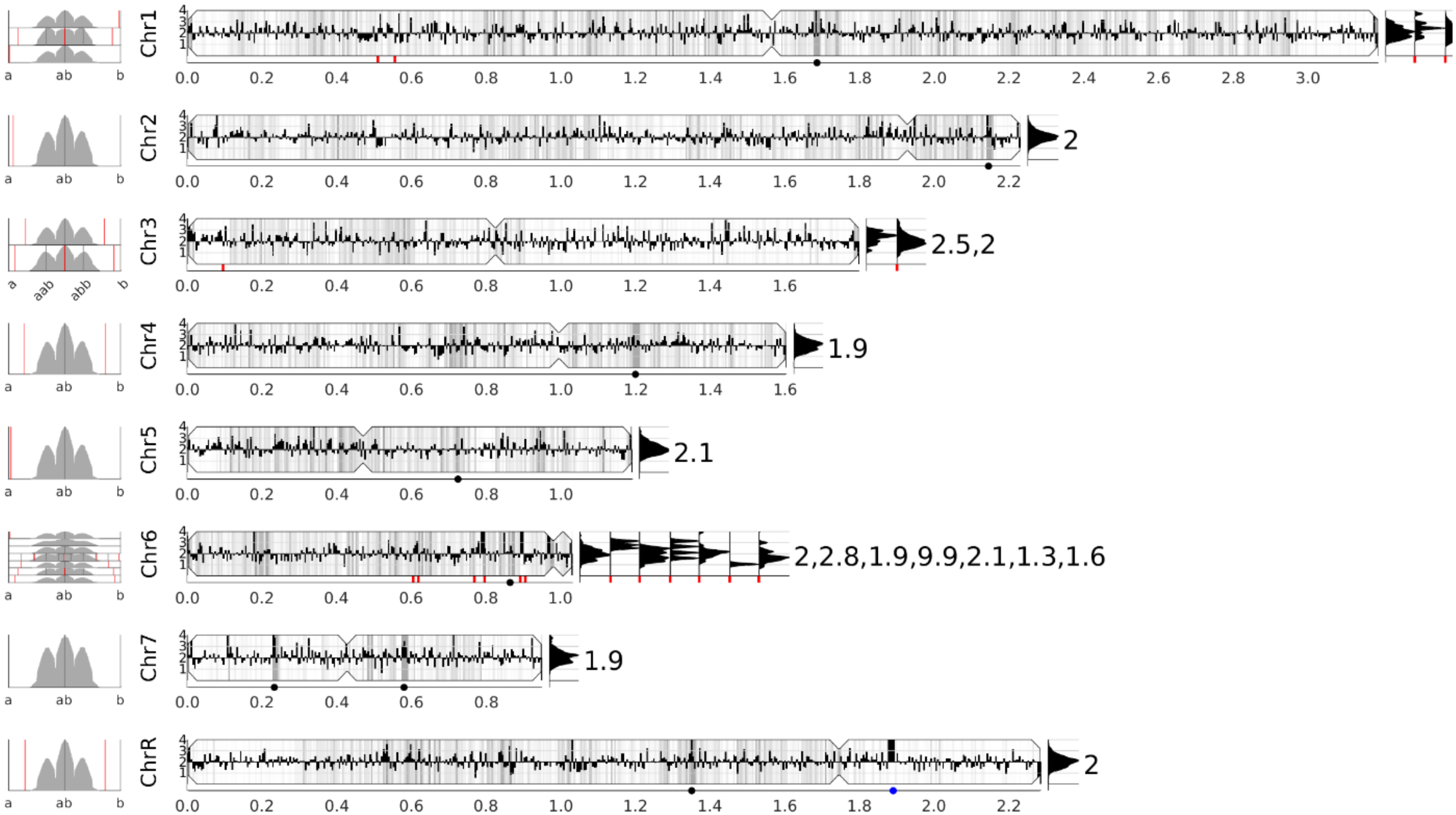

(D) Fluconazole-resistant isolate FL3: Compared to the control, FL3 maintained a largely stable diploid genome with preserved heterozygosity across all chromosomes. A localized copy number gain was observed on chromosome 4 and a loss on chromosome 5.

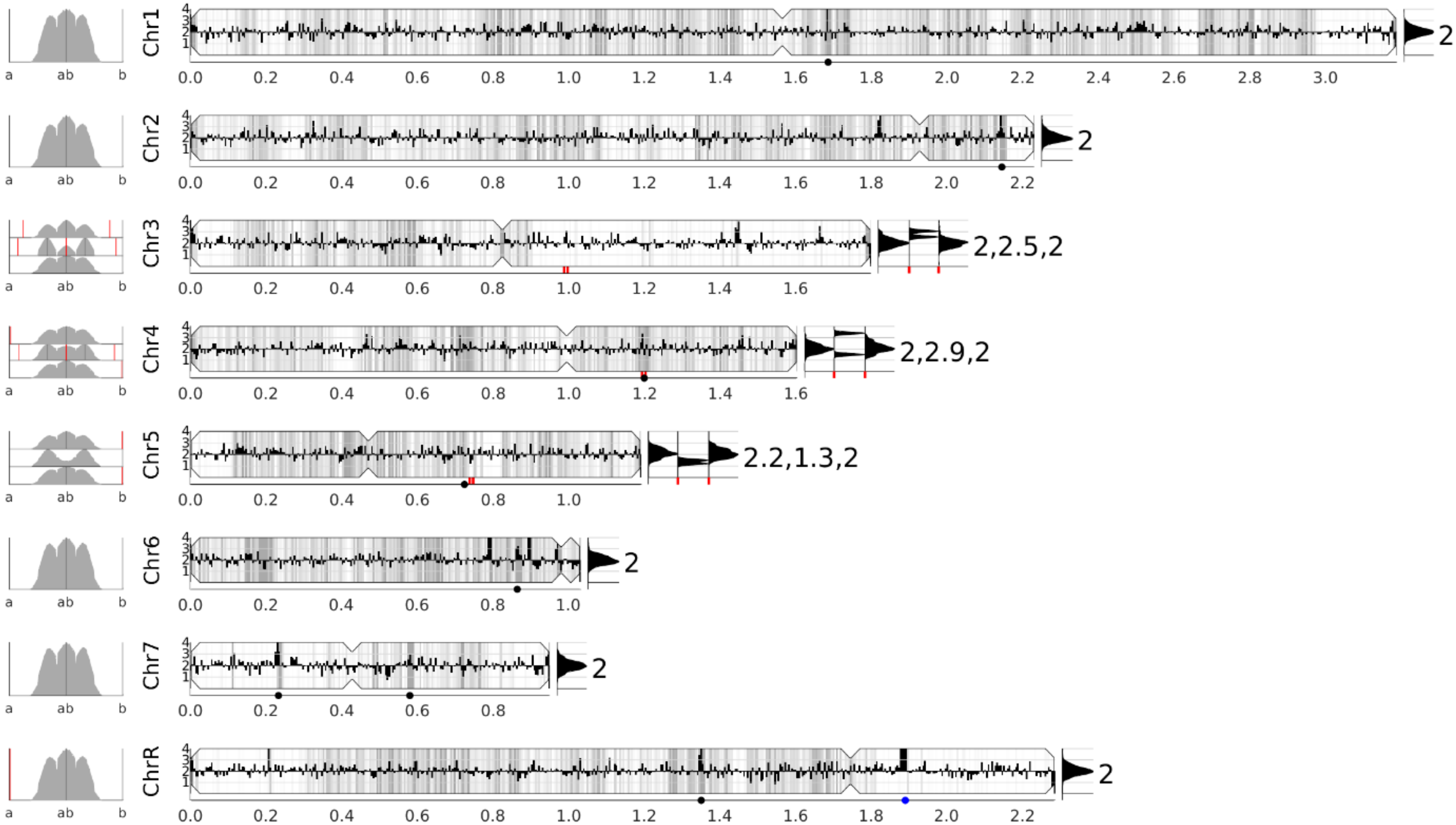

(E) Fluconazole-resistant isolate FL4: Compared to the control, FL4 maintained a largely stable diploid genome with preserved heterozygosity across all chromosomes.

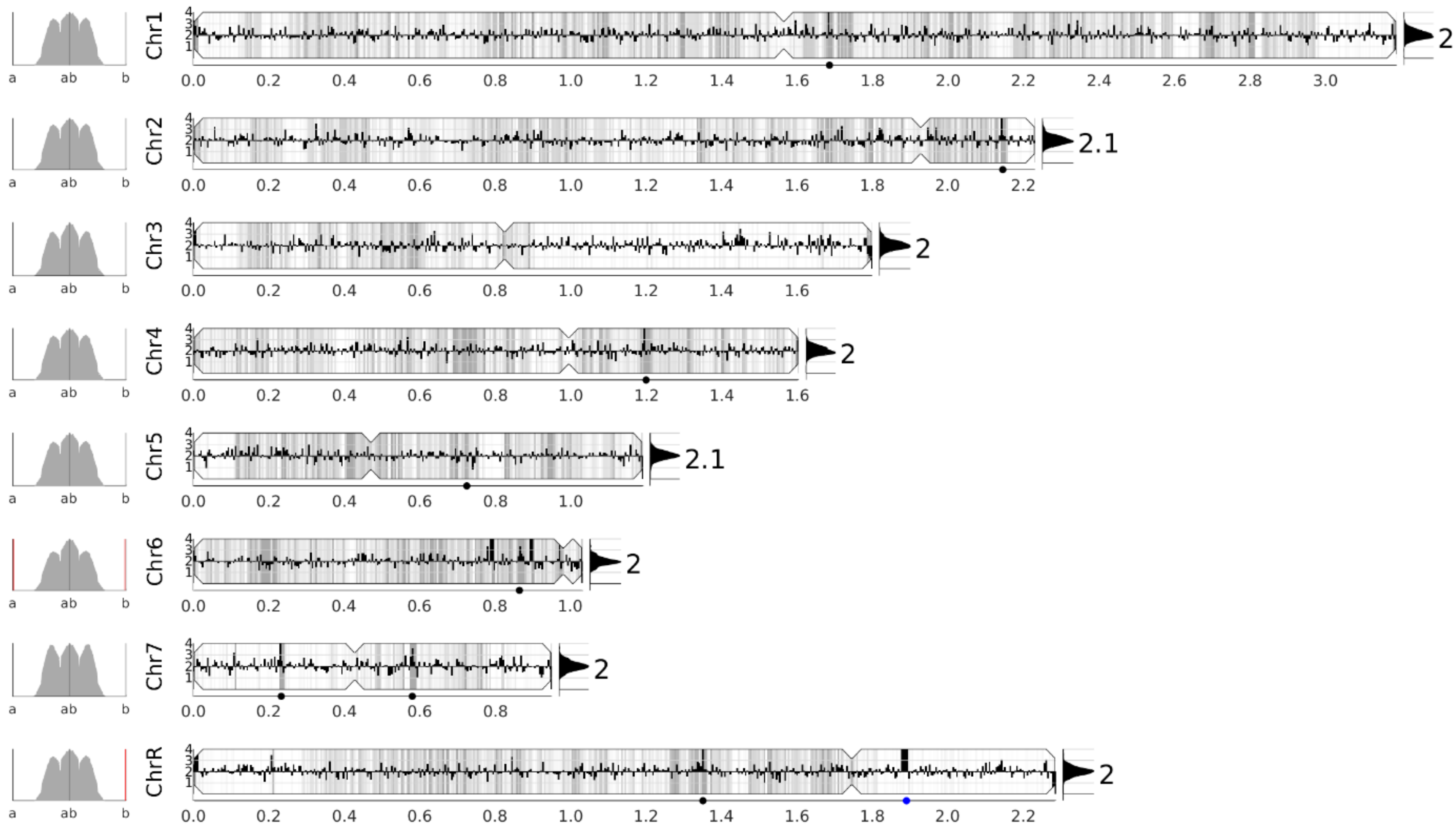

(F) Fluconazole-resistant isolate FL5: Compared to the control, FL5 showed widespread segmental copy number variation across multiple chromosomes while maintaining an overall diploid karyotype. The most extensive alterations were observed on chromosomes 5, 6 and 7, which contained several localized copy number gains and/or losses. Heterozygosity was largely preserved.

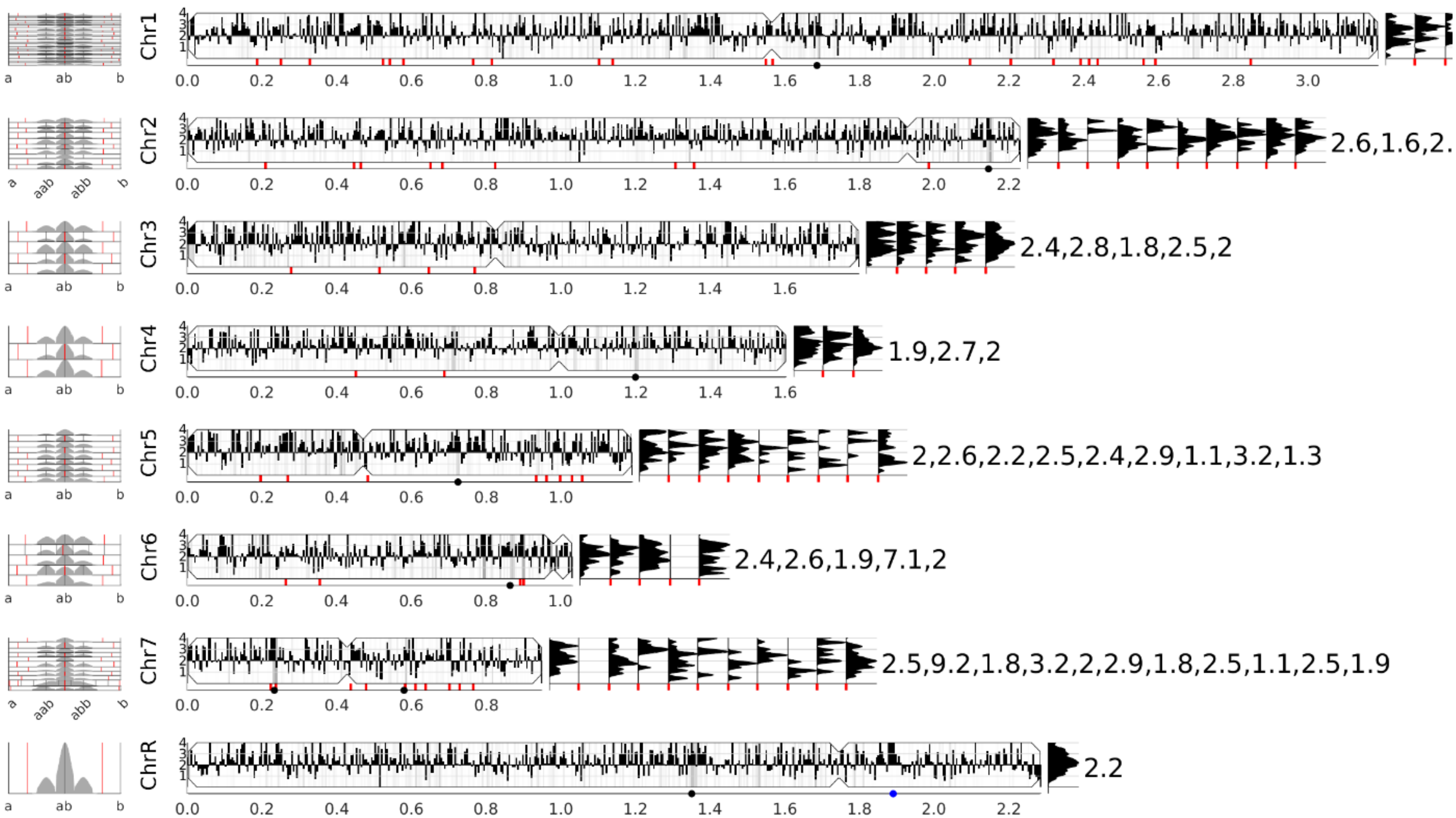

(G) Fluconazole-resistant isolate FL6: Compared to the control, FL6 showed widespread segmental copy number variation across multiple chromosomes while maintaining an overall diploid karyotype. The most extensive alterations were observed on chromosomes 3, 4 and 5, which contained several localized copy number gains and losses. Heterozygosity was largely preserved.

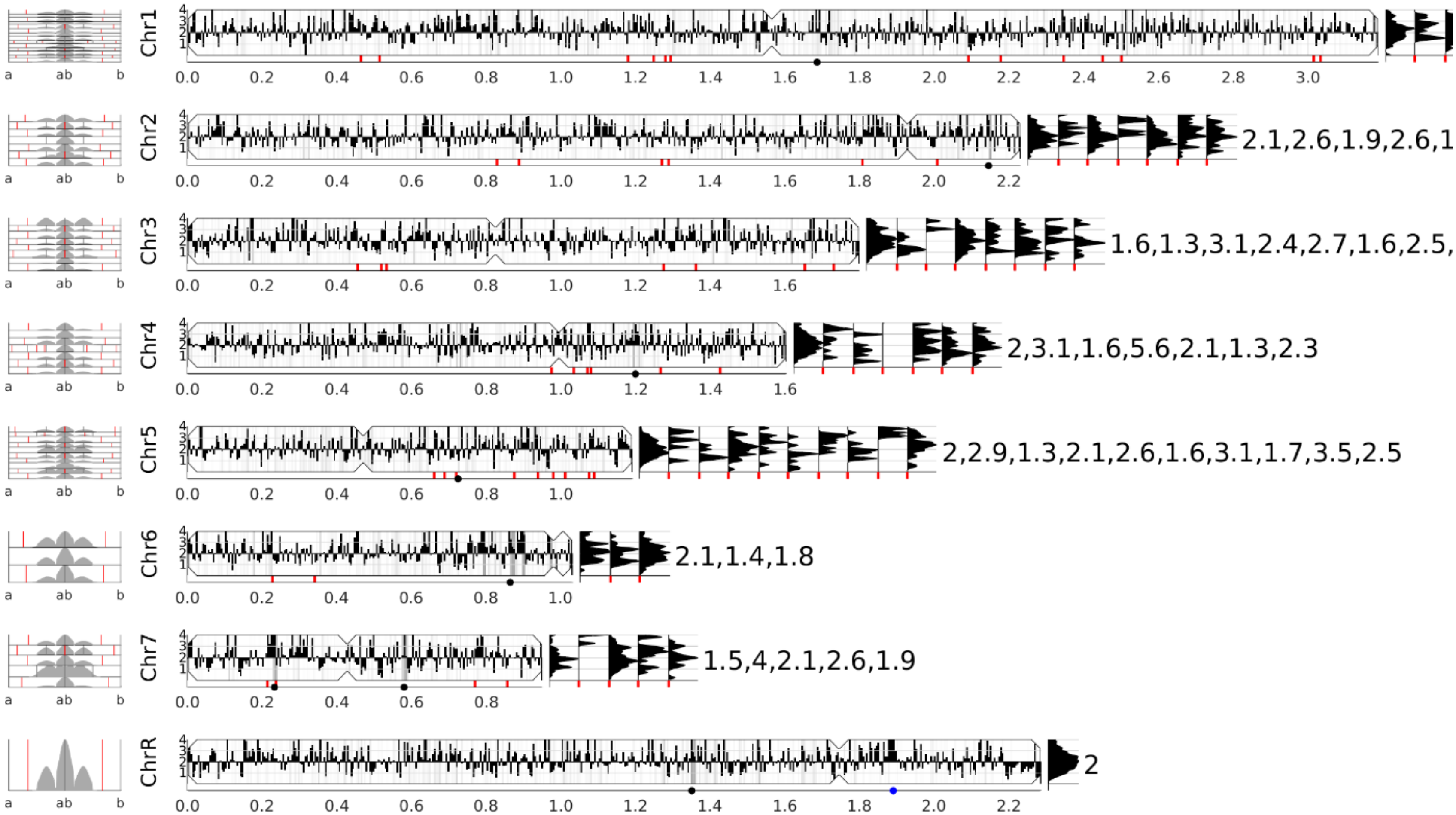

(H) Caspofungin-resistant isolate CS1: Compared to the control, CS1 showed localized segmental copy number variation across multiple chromosomes while maintaining an overall diploid karyotype. The most extensive alterations were observed on chromosomes 3, 4 and 5, which contained several localized copy number gains and/or losses. Heterozygosity was largely preserved.

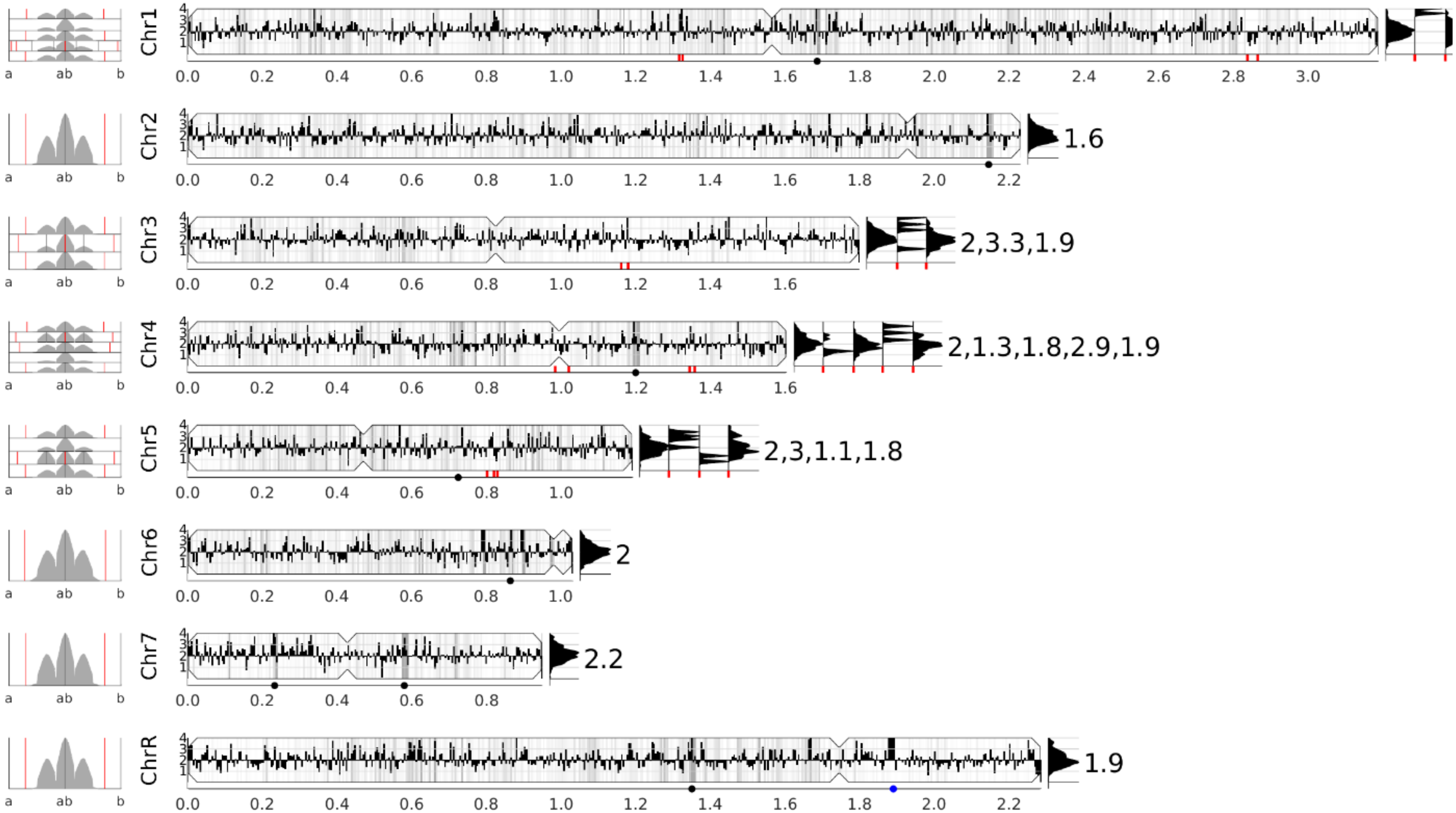

(I) Caspofungin-resistant isolate CS2: Compared to the control, CS2 showed widespread segmental copy number variation across multiple chromosomes while maintaining an overall diploid karyotype. The most extensive alterations were observed on chromosomes 3, 5, 6 and 7, which contained several localized copy number gains and losses. Heterozygosity was largely preserved.

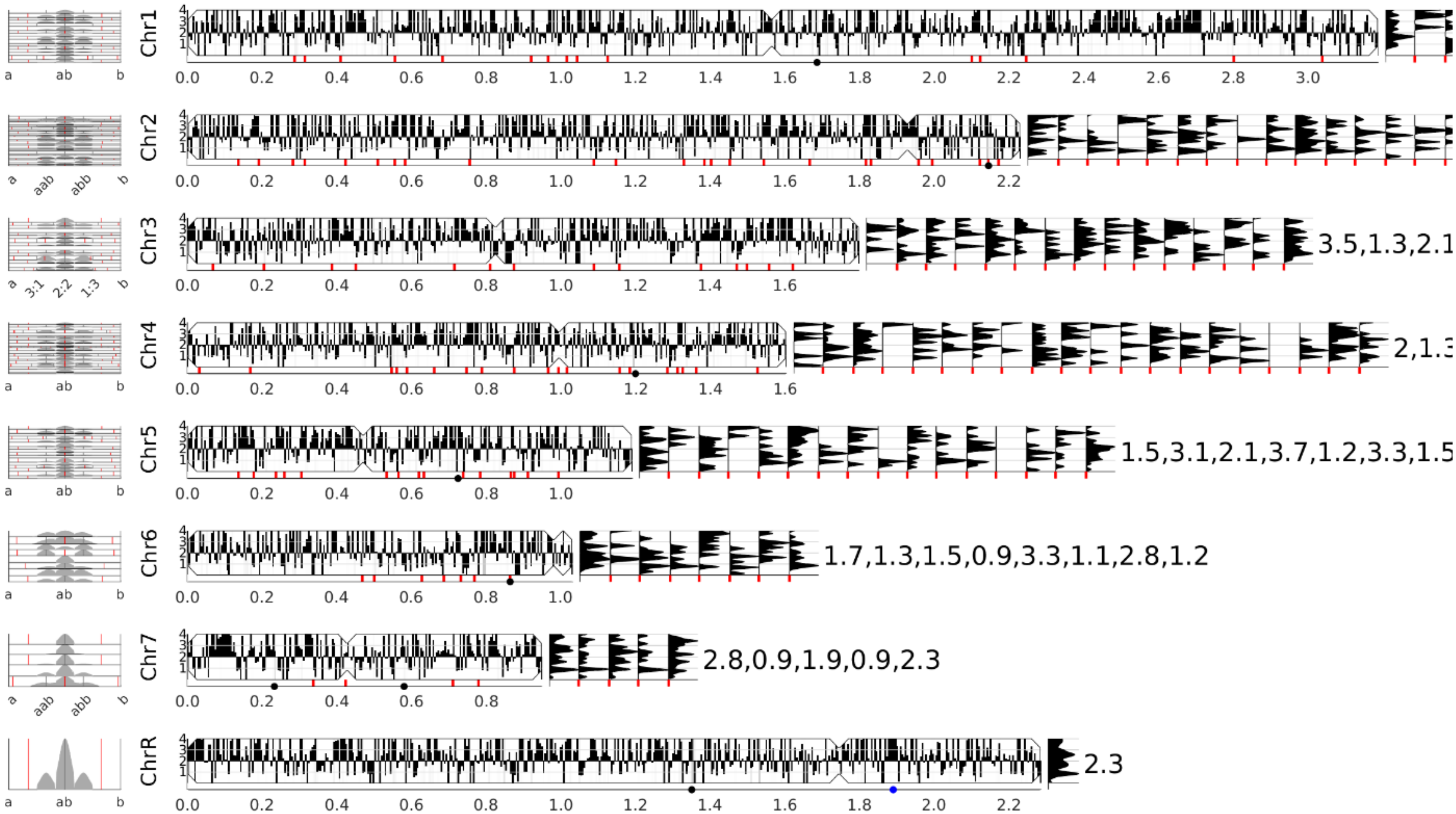

(J) Caspofungin-resistant isolate CS3: Compared to the control, CS3 maintained an overall diploid karyotype with limited genomic structural variation. The most notable alteration was a duplication on chromosome 3, while the remaining chromosomes displayed copy number profiles comparable to the control. Heterozygosity was largely preserved.

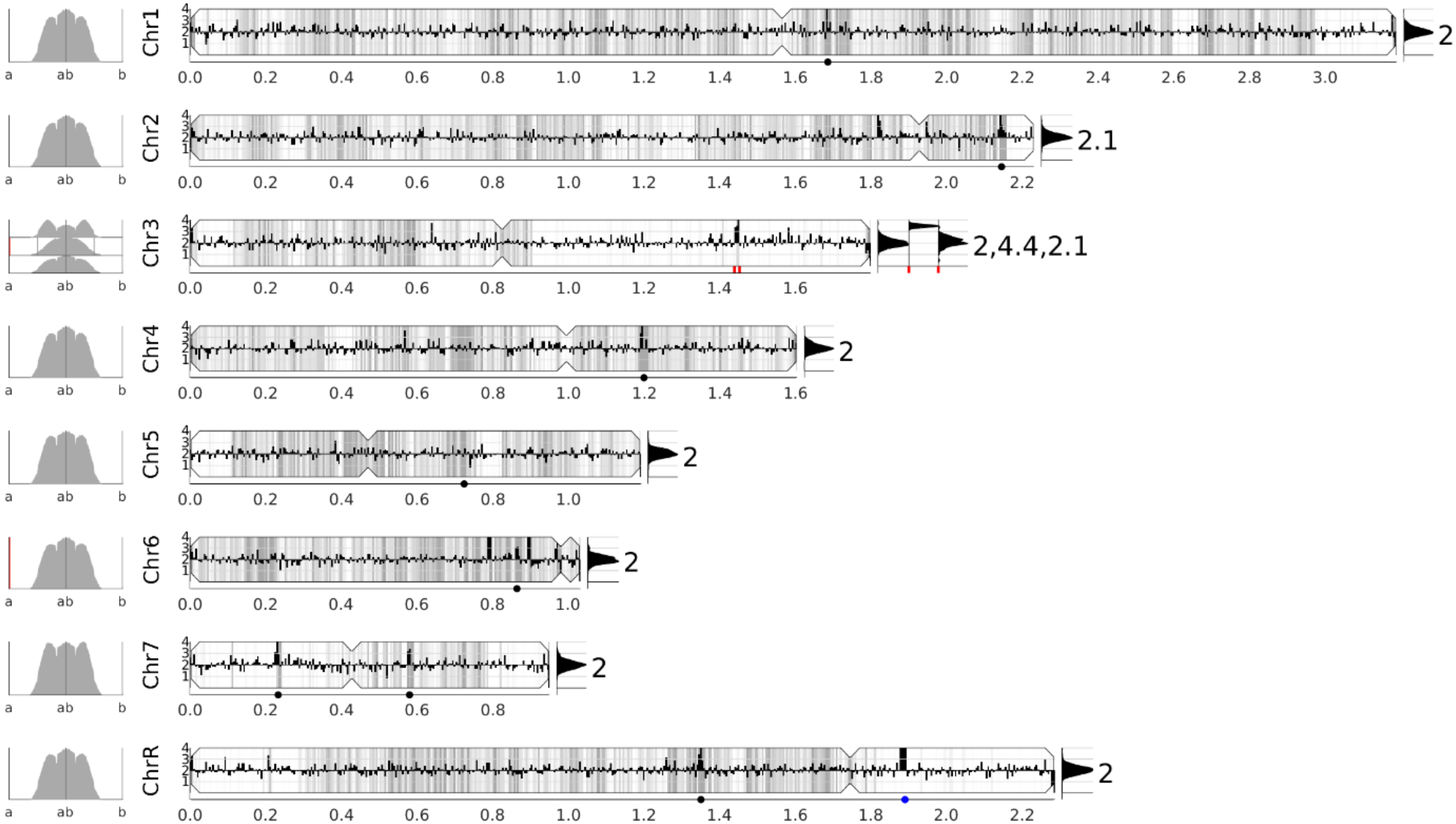

(K) Caspofungin-resistant isolate CS4: Compared to the control, CS4 maintained an overall diploid karyotype with limited genomic structural variation. The most notable alteration was a localized copy number gain on chromosome 4. Heterozygosity was largely preserved.

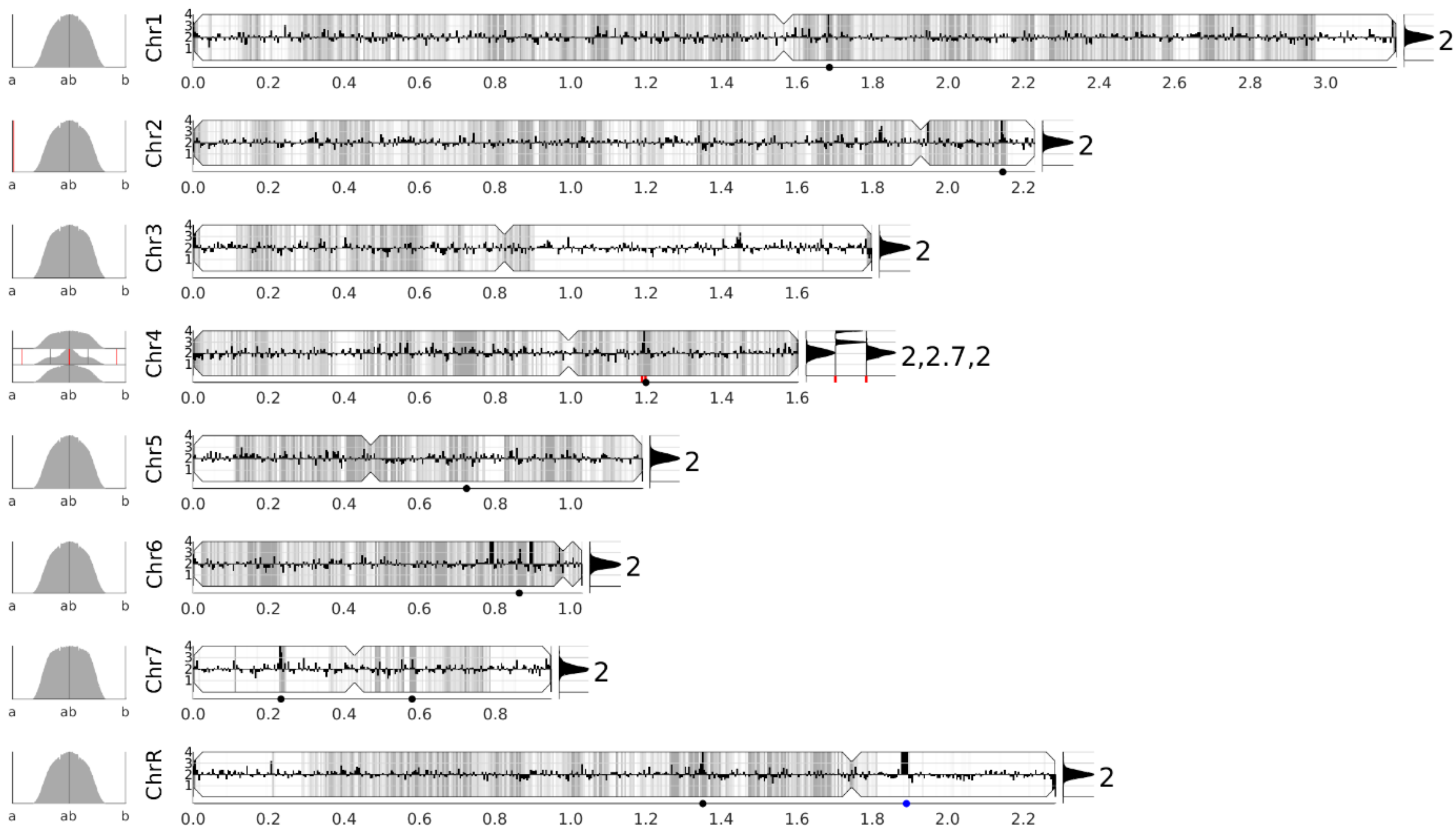

(L) Caspofungin-resistant isolate CS5: Compared to the control, CS5 showed localized segmental copy number variation across multiple chromosomes while maintaining an overall diploid karyotype. The most extensive alterations were observed on chromosomes 2 and 5 which experienced localized gains while chromosome 7 experienced a localized loss. Heterozygosity was largely preserved.

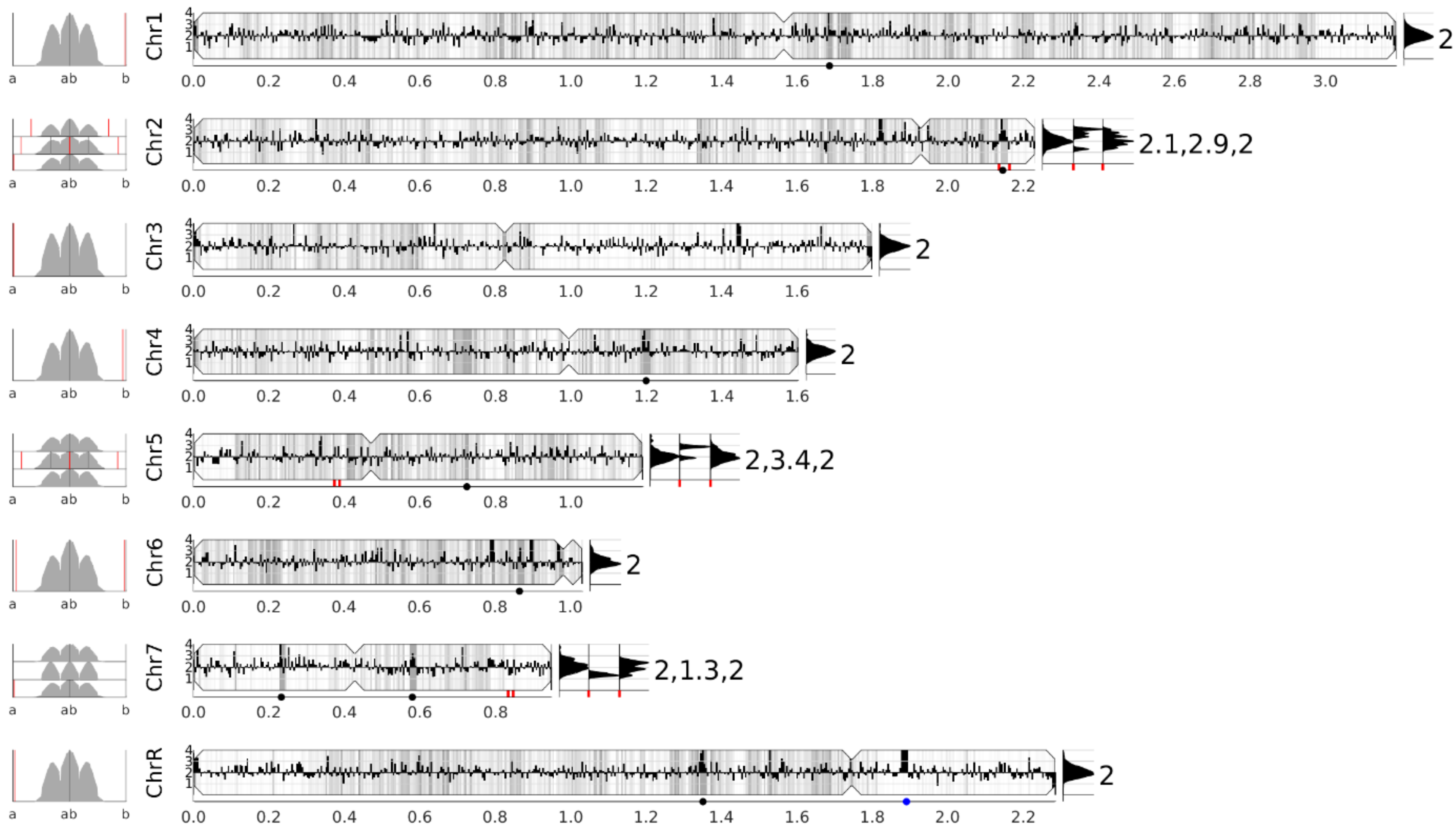

(M) Caspofungin-resistant isolate CS6: Compared to the control, CS6 showed localized segmental copy number variation across multiple chromosomes while maintaining an overall diploid karyotype. The most extensive alterations were observed on chromosomes 2 and 4 which experienced localized gains while chromosome 6 experienced a localized loss. Heterozygosity was largely preserved.

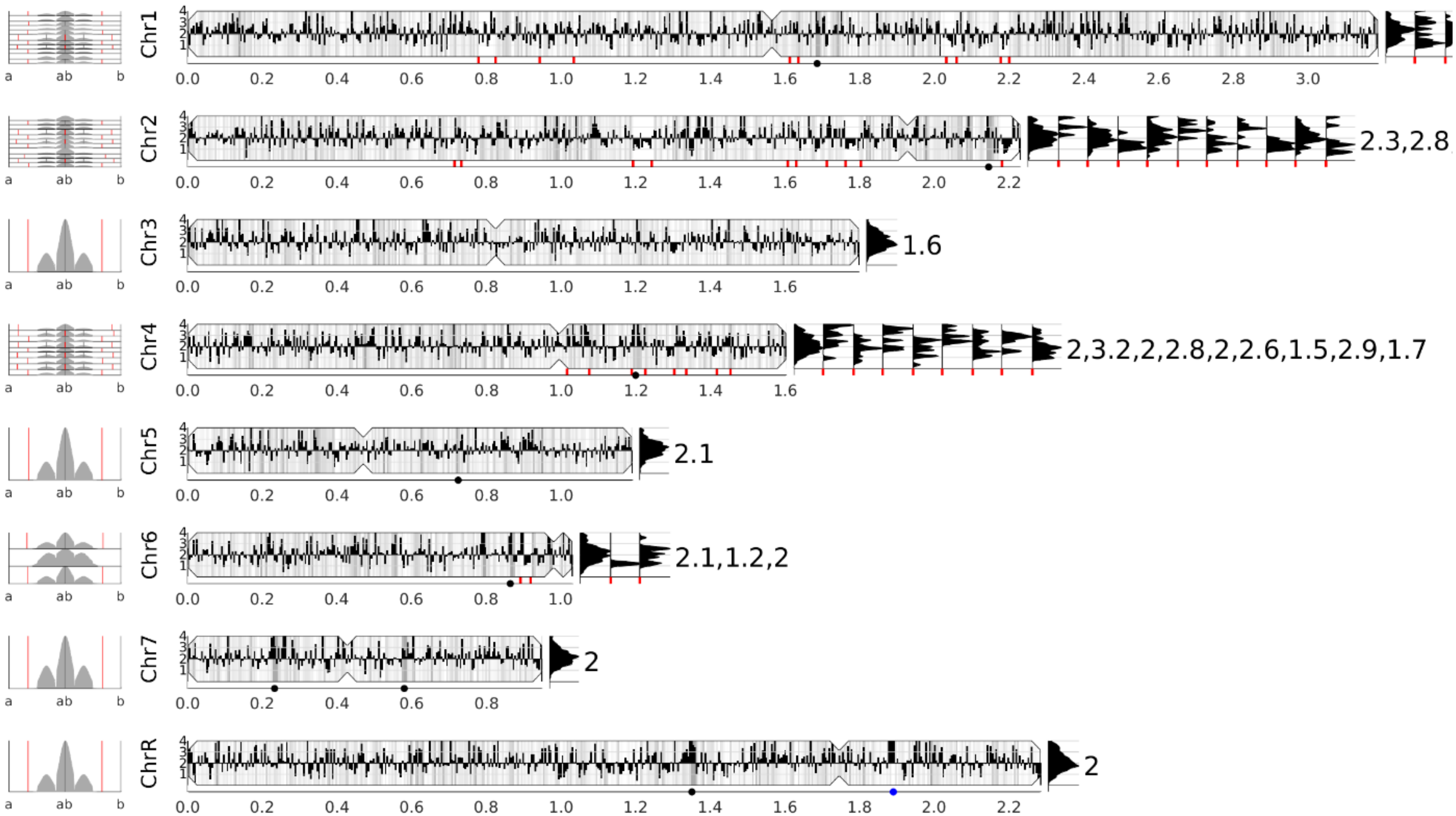

Supplement: Supplementary file 1 [file pone.0360234.s001.pdf]
